# Insights into the Genetic Structure and Diversity of 38 South Asian Indians from Deep Whole-Genome Sequencing

**DOI:** 10.1371/journal.pgen.1004377

**Published:** 2014-05-15

**Authors:** Lai-Ping Wong, Jason Kuan-Han Lai, Woei-Yuh Saw, Rick Twee-Hee Ong, Anthony Youzhi Cheng, Nisha Esakimuthu Pillai, Xuanyao Liu, Wenting Xu, Peng Chen, Jia-Nee Foo, Linda Wei-Lin Tan, Seok-Hwee Koo, Richie Soong, Markus Rene Wenk, Wei-Yen Lim, Chiea-Chuen Khor, Peter Little, Kee-Seng Chia, Yik-Ying Teo

**Affiliations:** 1 Saw Swee Hock School of Public Health, National University of Singapore, Singapore; 2 NUS Graduate School for Integrative Science and Engineering, National University of Singapore, Singapore; 3 Genome Institute of Singapore, Agency for Science, Technology and Research, Singapore; 4 Pharmacogenetics Laboratory, National University of Singapore, Singapore; 5 Cancer Science Institute of Singapore, National University of Singapore, Singapore; 6 Department of Biochemistry, National University of Singapore, Singapore; 7 Department of Biological Sciences, National University of Singapore, Singapore; 8 Life Sciences Institute, National University of Singapore, Singapore; 9 Department of Statistics and Applied Probability, National University of Singapore, Singapore; Dartmouth College, United States of America

## Abstract

South Asia possesses a significant amount of genetic diversity due to considerable intergroup differences in culture and language. There have been numerous reports on the genetic structure of Asian Indians, although these have mostly relied on genotyping microarrays or targeted sequencing of the mitochondria and Y chromosomes. Asian Indians in Singapore are primarily descendants of immigrants from Dravidian-language–speaking states in south India, and 38 individuals from the general population underwent deep whole-genome sequencing with a target coverage of 30X as part of the Singapore Sequencing Indian Project (SSIP). The genetic structure and diversity of these samples were compared against samples from the Singapore Sequencing Malay Project and populations in Phase 1 of the 1,000 Genomes Project (1 KGP). SSIP samples exhibited greater intra-population genetic diversity and possessed higher heterozygous-to-homozygous genotype ratio than other Asian populations. When compared against a panel of well-defined Asian Indians, the genetic makeup of the SSIP samples was closely related to South Indians. However, even though the SSIP samples clustered distinctly from the Europeans in the global population structure analysis with autosomal SNPs, eight samples were assigned to mitochondrial haplogroups that were predominantly present in Europeans and possessed higher European admixture than the remaining samples. An analysis of the relative relatedness between SSIP with two archaic hominins (Denisovan, Neanderthal) identified higher ancient admixture in East Asian populations than in SSIP. The data resource for these samples is publicly available and is expected to serve as a valuable complement to the South Asian samples in Phase 3 of 1 KGP.

## Author Summary

Indians of South Asia has long been a population of interest to a wide audience, due to its unique diversity. We have deep-sequenced 38 individuals of Indian descent residing in Singapore (SSIP) in an effort to illustrate their diversity from a whole-genome standpoint. Indeed, among Asians in our population panel, SSIP was most diverse, followed by the Malays in Singapore (SSMP). Their diversity is further observed in the population's chromosome Y haplogroup and mitochondria haplogroup profiles; individuals with European-dominant haplogroups had greater proportion of European admixture. Among variants (single nucleotide polymorphism and small insertions/deletions) discovered in SSIP, 21.69% were novel with respect to previous sequencing projects. In addition, some 14 loss-of-function variants (LOFs) were associated to cancer, Type II diabetes, and cholesterol levels. Finally, D statistic test with ancient hominids concurred that there was gene flow to East Asians compared to South Asians.

## Introduction

Next-generation sequencing (NGS) technologies have enabled an entire genome to be sequenced in a cost-effective manner [1], and this has allowed multiple individuals from a population to be surveyed in order to catalogue genetic variants that are present in the population. By adopting an unbiased approach to survey the whole genome, NGS presents a more comprehensive catalogue of different classes of genetic variants with a single assay: from changes that affect only a single base in the genome (single nucleotide polymorphisms, SNPs), to small-size additions and omissions (insertion-deletions, indels); and to larger contiguous changes in the genome that affect either the number of copies of a stretch of genome or differences in the genomic structure (structural variants, SVs). Prior to the advent of NGS, the knowledge of these different classes of variants at the population level has been derived primarily from surveys using pre-designed microarrays, and where the majority focused on SNP variations between populations.

The 1000 Genomes Project (1 KGP) intends to survey more than 2,500 individuals from at least 20 populations around the world [2], and Phase 1 of the project has already offered valuable insights into the population genetics of 14 populations. While the 1 KGP adopted the approach of sequencing multiple individuals at a lower sequence depth of 2-6X, a recently concluded project in Southeast Asia sequenced 100 Austronesian Malays (the Singapore Sequencing Malay Project, SSMP) at a coverage of at least 30X examined the merits of deep sequencing for a more complete characterization of variants carried by the individuals [3]. The use of NGS has also been successfully extended to sequence ancient hominids such as the Neanderthals and the Denisovans [4], [5], [6], and from well-preserved human tissues from the Ötzi [7] and an aboriginal Australian [8].

South Asia comprises more than 20% of the total world population, of which the majority resides in India [8]. The contemporary demographic makeup of the Indian subcontinent is considerably heterogeneous and is the result of complex human migration and interaction since the first human dispersal out of Africa between 60,000 to 75,000 years ago. It was first proposed from the analysis of mitochondria sequences that a single rapid coastal dispersion happened from the Horn of Africa into Southeast Asia and Australasia through the Indian subcontinent [9], and which corroborated with additional analyses of mitochondria in the Andaman and Nicobar Islands [10]. Genome-wide genotyping surveys of geographically well-defined South Asians indicated the presence of complex admixture between populations in the Indian subcontinent [11], [12], which have been proposed to be attributed to the practice of the caste system which encouraged endogamous marriages and the presence of different ethno-linguistic groups – the Indo-Aryan language speaking groups that are primarily found in north India, and the Dravidian language speaking groups that are predominantly found in south India.

Singapore is home to more than 350,000 Indians, comprising 9.2% of the residing population [13]. These people with ancestry originating from the Indian subcontinent has been designated officially and uniformly as “Indian”, although this can be inadequate given the heterogeneous background of the Indian populace in Singapore, which includes Tamils, Malayalee, Sikh, Hindustani, Punjabi, Sindhi, Hindi, Gujarati, Urdu and Sinhalese [14]. The majority of the Singapore Indians were descended from immigrants from south India who settled in the country after Singapore became a major entrepot trading center in the early 19^th^ century [15], [16].

The SSIP aims to perform whole-genome sequencing of 38 healthy Singapore Indians, to provide insights into the genetic structure and diversity of Asian Indians in Singapore. With a target sequence depth of 30X, the SSIP presents another public resource of deep whole genome sequencing of multiple samples in a well-defined population, as with the SSMP. Here, we evaluated the genetic proximity of the SSIP to the SSMP and 14 populations from Phase 1 of the 1 KGP, and measured the degree of intra-population genetic diversity in each of the 16 populations. The ancestral origins of the SSIP samples were inferred by evaluating the mitochondria and chromosome Y haplogroup memberships of the relevant samples, and we performed an analysis of the relative relatedness between SSIP and two archaic hominins (Denisovan, Neanderthal). The genetic resource for the SSIP is publicly available at http://www.statgen.nus.edu.sg/~SSIP.

## Results

### Sequence data assembly, alignment and quality control

South Asian Indians from the Singapore Population Health Study were sampled to be whole-genome sequenced with the Illumina HiSeq 2000 to a target 30-fold coverage, using paired-end sequencing with 100 basepairs (bp) reads and a target insert size of between 300 bp and 400 bp (Figure S1). This was performed on 38 subjects, comprising 26 females and 12 males, where ethnic membership for each sample was confirmed through verbal reconfirmation that all four grandparents were similarly of South Asian descent. A total of 56 billion paired-end reads were generated for the 38 samples, of which 42.7 billion reads were properly paired and passed quality assessment (Figure S2). Sequence reads were mapped to the NCBI build 37 reference genome with Consensus Assessment of Sequence And VAriation (CASAVA v1.9), and variant calling for SNPs and indels were performed with both the single-sample caller CASAVA and the multi-sample caller Genome Analysis Toolkit (GATK) (Figure S3). All but one of the 38 SSIP samples (SSI007) achieved median sequence depths greater than 30X (Figure S4). To assess the accuracy of the self-reported population membership, a principal component analysis (PCA) was carried out on the 38 SSIP samples and 268 samples from the Singapore Genome Variation Project (SGVP), where one sample (SSI016) clearly clustered with the Malays instead of the Indians (Figure S5). We thus excluded both SSI007 and SSI016 from further analyses, and the remaining samples consisted of 25 females and 11 males.

### SNP discovery and annotation

The data release for SSIP consisted of the consensus set of SNPs and indels called by both CASAVA and GATK to minimize false discovery. As each sample was also genotyped on the Illumina Omni2.5 M microarray, we compared the concordance of the genotype calls made by CASAVA and GATK with the genotypes at the same SNPs on the Omni2.5 M. CASAVA was observed to produce genotype calls with a higher concordance with the Omni2.5 M genotypes than GATK (Figure S6), we subsequently retained the CASAVA genotypes release in the SSIP VCFs and downstream analyses.

**Figure 1:**
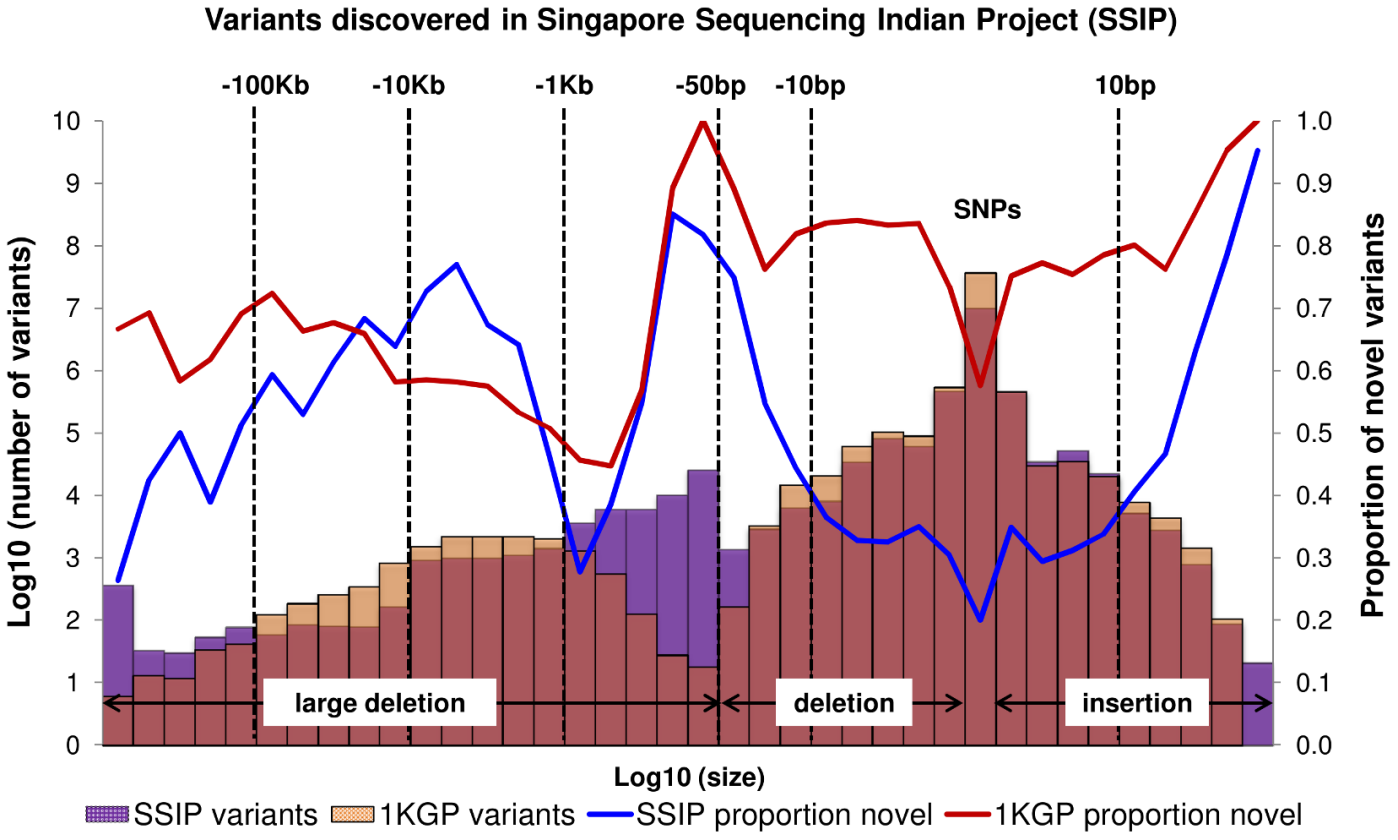
Size distribution and novelty of variants in SSIP. Autosomal variants identified in the 36 SSIP samples, which included single nucleotide polymorphisms (SNPs), small insertion/deletions (indels) between 2 bp to 50 bp, and large deletions between 51 bp to 1 Mb. The SSIP SNPs and indels are defined as novel if they are not present in SSMP and dbSNP137, whereas dbSNP132 was used for defining the novelty of the 1 KGP SNPs and indels. The novelty of large deletions in SSIP and 1 KGP is defined with respect to SSMP and DGV release 2013-07-23.

A total of 10,305,409 SNPs, 1,269,000 indels and 56,088 large deletions were detected in the 36 SSIP samples (Table 1), where 20.02%, 32.90% and 76.67% respectively were found to be novel (defined as not present in dbSNP137, 1 KGP, the SSMP or the Database of Genomic Variants (DGV) accordingly, Figure 1). While the vast majority of the SNPs discovered were bi-allelic, there were 18,904 tri-allelic and 27 quad-allelic SNPs, of which 5.4% of these were not previously known. The transition-to-transversion (Ts/Tv) ratio for the bi-allelic SNPs was 2.14 across the whole genome, and was significantly higher at 3.24 if restricted to only the exonic SNPs (Table S3) due to the over-representation of CpG sites in the exome (calculated in SSIP at 32.6% in the exome, and 17.2% across the genome). With the available sample size, each variant is classified according to the alternative allele frequency (AAF) as either low-frequency (AAF<5%) or common (AAF≥5%). The majority of the known bi-allelic SNPs were common while the opposite was observed for novel SNPs, where the majority was low-frequency in nature (Table 1).

**Table 1:** Summary of variants discovered in SSIP.

	NOVEL*			KNOWN			ALL		
	**Low**	**Common**	**Subtotal**	**Low**	**Common**	**Subtotal**	**Low**	**Common**	**Total**
**Bi-allelic SNPs**	2,036,480	54,888	2,091,368	2,204,240	5,990,870	8,195,110	4,240,720	6,045,758	10,286,478
**High**	323	3	326	260	342	602	583	345	928
**Moderate**	9,967	115	10,082	11,120	16,176	27,296	21,087	16,291	37,378
**Low**	7,337	96	7,433	10,369	20,467	30,836	17,706	20,563	38,269
**Modifier**	2,018,853	54,674	2,073,527	2,182,491	5,953,885	8,136,376	4,201,344	6,008,559	10,209,903
**SIFT_PP2 Damage**	2,162	17	2,179	1,762	1,134	2,896	3,924	1,151	5,075
**Average number of SNPs per sample**					3,308,876				
**Indels**	33	417,117	417,150	17	851,833	851,850	50	1,268,950	1,269,000
**High**	0	334	334	0	378	378	0	712	712
**Moderate**	0	247	247	0	343	343	0	590	590
**Low**	0	0	0	0	0	0	0	0	0
**Modifier**	33	416,536	416,569	17	851,112	851,129	50	1,267,648	1,267,698
**Average number of indels per sample**					395,224				
**Structural variation (deletion)**	26,579	15,300	41,879	3,529	10,680	14,209	30,108	25,980	56,088
**Average number of SV per sample**					7,713				

*Definition of novel SNPs and indels is made with reference to known variants in dbSNP137 and SSMP, while definition of novel structural variants is made with reference to known variants in Phase 1 of 1 KGP, SSMP and DGV.

Bi-allelic SNPs and indels were further annotated with SNPEff which catalogued each variant into one of four categories according to the predicted level of impact to protein function [17]. In the SSIP samples, 928 and 37,378 SNPs were classified into the high and moderate impact categories respectively (Table 1). Among these SNPs, 5,075 SNPs were predicted by both SIFT and PolyPhen v2 to be damaging to the protein product. Similarly, the majority of the indels were catalogued as modifiers with low or no impact to protein function, and only 712 and 590 indels were annotated as high and moderate impact variants respectively. Intriguingly, almost all the observed indels were present in the SSIP samples at minor allele frequencies >5%.

When compared to the variants that have been reported by 1 KGP, there were more SNPs and indels present in 1 KGP than SSIP (Figure 1). However, SSIP identified more deletions between the sizes of 50 bp and 1 kb than 1 KGP. This is likely attributed to the higher coverage of the sequencing, which provided greater confidence in identifying the smaller deletions that may only be identified with sufficient sequence depth.

### Loss-of-function variants

We identified 1,429 loss-of-function variants (LOFs) in the 36 SSIP samples, of which 635 LOFs were novel and 407 LOFs had AAF>5% (Table S4). On average, each sample carried 350 LOFs, which exceeded a previous report of around 100 genuine LOFs per healthy individual [18] but was similar to the average of 470 LOFs per sample in the SSMP [3]. A gene-set analysis using Visualization and Integrated Discovery (DAVID) identified significant enrichment of LOFs in pathways related to olfactory transduction, ATP-bind cassette (ABC) transporters and Histidine metabolism, although only the olfactory transduction pathway remained statistically significant after correcting for multiple testing with the Benjamini-Hochberg procedure (*P*_corrected_ = 2.8×10^−3^, Table S5). The LOFs in the olfactory pathway may have emerged to perceive chemicals due to differential diet and environmental exposure that may have geographical specificity.

The LOFs were also mapped to the Catalogue of Somatic Mutations In Cancer (COSMIC) database with SNPnexus, and 11 variants were found to be associated with ovarian, gastrointestinal, hepatic and pancreatic cancer (Table S6). The risk alleles at these variants generally were found at higher frequencies in the SSIP samples, although we caution against over-interpreting the significance given the small sample set in the SSIP. When checked against the genome-wide association studies (GWAS) database, 3 LOFs were reported to be associated with conduct disorder, triglyceride and high density lipoprotein cholesterol, and Type 2 diabetes (Table S7). In particular, the LOF rs1048886 that was reported to be associated with diabetes was first established from a diabetes GWAS in Singapore Indians.

### Population structure of SSIP samples

**Figure 2:**
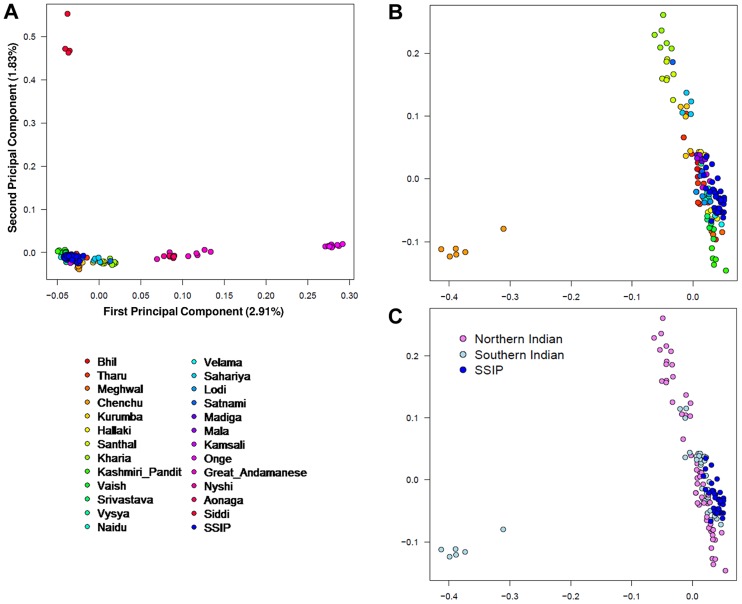
Principal component analysis (PCA) of SSIP samples with 132 South Asians. PCA of 36 SSIP samples with 132 South Asian samples from 25 well-defined Indian groups by Reich and colleagues [44] using 202,600 SNPs that were present in both databases (panel A). Five groups corresponding to Great Andamanese, Onge, Nyshi, Aonaga and Siddi were subsequently removed, leaving 104 samples from 20 Indian groups to be analyzed in a second PCA, where the samples were first assigned a color according to their group memberships (panel B), and second by the latitude of origin into North and South Indians (panel C, see Table S2 for the classification of North and South Indians). The color assignments in panels A and B are represented by the color legend on the bottom left of the figure.

A PCA was performed with the SSIP samples and a panel of 132 South Asians from 25 well-defined groups from the Indian subcontinent [11] in an attempt to understand the ancestral origins of the Singapore Indians. Consistent with the findings by Reich and colleagues [11], the first principal component (PC) distinguished the Great Andamanese, Onge, Nyshi, AoNaga and Siddi samples from all other Indian subgroups (including SSIP, Figure 2A). When samples from these five groups were removed, it was observed that SSIP samples were located with all the south Indian groups (Hallaki, Kamsali, Velama) except Chenchu (Figures 2B, C), although there were considerable heterogeneity amongst the north Indian groups (Figure 2C) that precluded a clear latitudinal distinction of north and south Indians. We performed admixture analysis on 104 individuals from 20 Indians groups reported by Reich and colleagues, together with the SSIP samples. The results supported the hypothesis that the SSIP individuals are genetically more homogenous to the south Indians than to the north Indians (Figure S14).

**Figure 3:**
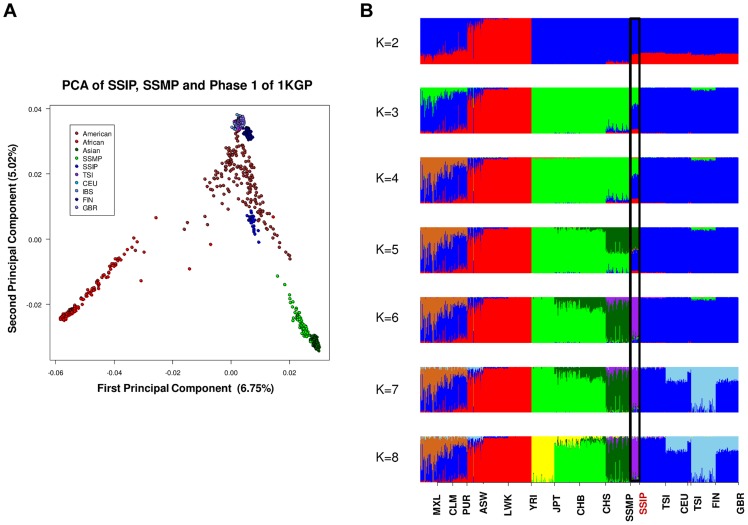
Principal component analysis (PCA) of 1,224 samples from 16 global populations. PCA of 1,224 samples from SSIP, SSMP and 14 populations from Phase 1 of the 1-coded by continents (panel A). An analysis of admixture was also performed on the 16 populations with ADMIXTURE, where the number of distinct populations (*K*) was allowed to vary between 2 and 8 (panel B). The black window highlights the position of the SSIP samples on the admixture plot.

A separate PCA with samples from 16 populations (SSIP, SSMP and Phase 1 of 1 KGP) was performed to place these 36 Singapore Indians amongst global populations (Figure 3A). The first PC differentiated the SSIP samples from Africans and East Asians although the second PC was necessary to distinguished them from the Europeans. However, there appeared to be considerable diversity between the Americans that confounded the distinction between them and the SSIP samples. This was similarly evident in the F_ST_ analyses, where the Americans were found to be most similar to the SSIP (Figure S7). Excluding the admixed American populations, the SSIP was genetically closest to the Europeans in the panel of populations considered.

In an admixture analysis of the 16 populations, we observed that the SSIP were consistently inferred to be significantly admixed with the Europeans until at least six distinct groups were assumed (*K*≥6, Figure 3B), where the European component stabilized to an average of 7.1%, 4.3% of Malay and 1.3% of East Asians (Table S8).

### Assessing between and within population diversity

**Figure 4:**
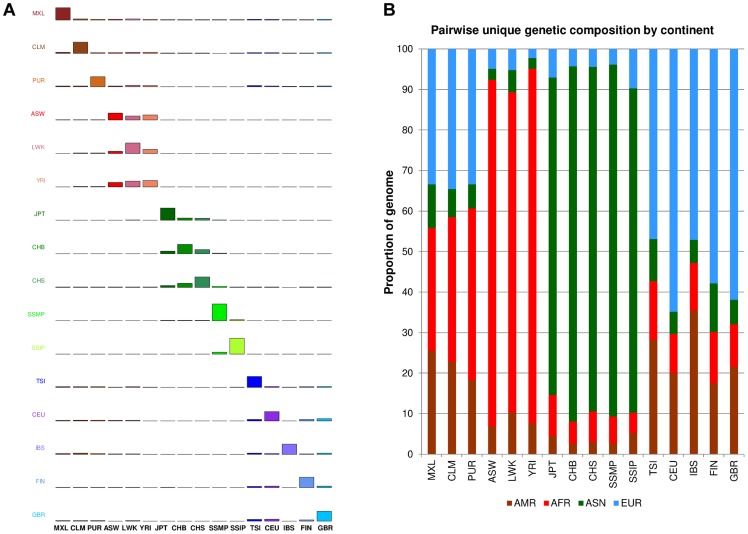
Unique SNP sharing between populations. (A) Each row represents the distribution of SNPs that are shared uniquely between a reference population (vertical axis) and a target population (horizontal), where the bars along the diagonal indicate the number of SNPs that are unique to the reference population. Here, unique sharing is defined as SNPs that are present only in the two respective populations but not others. (B) Distribution of SNPs in the reference population (horizontal) that are shared by only one other population, but here the target populations are grouped by continents into four broad categories of the Americas (AMR: CLM, MXL, PUR), Africans (AFR: ASW, LWK, YRI), Asian (ASN: CHB, CHS, JPT, SSMP, SSIP) and Europeans (EUR: CEU, FIN, GBR, IBS, TSI).

One measure of population diversity we investigated was the proportion of SNPs that was shared uniquely with only one other population when assessed across all 16 populations (Figure 4A). We observed that SSIP exhibited the greatest sharing with SSMP, although this is likely to be attributed to the >30X sequencing depth for both populations, since the remaining 14 populations in 1 KGP were sequenced at between 2-6X. Unsurprisingly, populations from the same continent generally exhibited higher sharing amongst themselves (Figure 4B) and the admixed populations from the Americas tend to possess greater sharing with either the European populations or the African populations.

**Figure 5:**
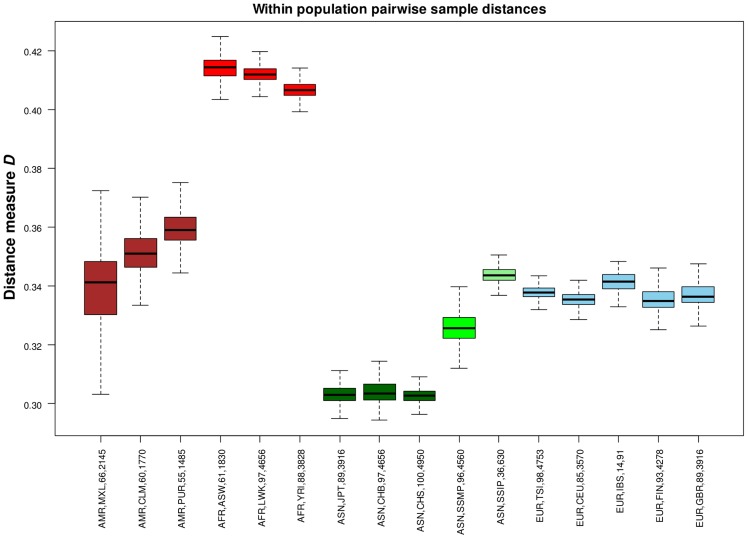
Assessing intra-population diversity between the samples. The extent of SNP sharing between every pair of samples in a population can be measured with a distance measure *D* that is scaled between 0 and 1 (vertical axis), where a higher value indicates a greater extent of heterogeneity in SNP content (or a lower degree of SNP sharing) between two samples. All possible pairwise measurements of *D* in each population are represented in a boxplot, where the ends of the whiskers indicate the minimum and maximum distances between specific pairs of samples in that population, the edges of the box indicates the 1^st^ and 3^rd^ quartiles, and the horizontal line in the box represents the median pairwise distance. The groups are colored with respect to the four continents (Americas – maroon; Africans – red; Asians – green; Europeans – blue). Each label on the horizontal axis indicates the continent label, population label, number of samples and total number of sample pairs of the population.

In addition to evaluating the inter-population diversity, we also investigated intra-population diversity by measuring the degree of SNP sharing between every pair of samples in each population. This presented a distance measure *D* that is scaled between 0 and 1, where a higher value indicated a greater degree of heterogeneity in SNP content between the two samples (or a lower degree of SNP sharing). As expected, the African populations exhibited the highest intra-population diversity while the East Asian populations exhibited the lowest (Figure 5). In comparison, SSIP had a median intra-population diversity that was marginally higher than the Europeans, while SSMP was between the East Asians and Europeans. Other than the median, the spread of the *D* metric calculation between every pair of samples is also indicative of the inter-sample diversity within a population. As expected, the American populations exhibited the largest spread due to differential degree of admixture between the samples. Excluding the Americans, SSMP exhibited the largest spread for a population, suggesting that there were considerable differences in the extent of genetic dissimilarities between the Singapore Malays, likely a reflection of the heterogeneous ancestry of the Singapore Malays. The trend in the distribution of the *D* metric was consistent even when the analysis was restricted to 36 samples from each population (Figure S8) to avoid confounding due to the different number of samples in each population. Consistent results were similarly observed when the same analysis was performed on samples from seven populations in 1 KGP that were sequenced by Complete Genomics to deeper coverage of between 51X and 89X, which included the Gujarati Indians from Houston that exhibited a *D* distribution highly concordant to the SSIP (Figure S9).

While SSIP exhibited the highest intra-population diversity amongst the five Asian populations (CHB, CHS, JPT, SSMP, SSIP), we similarly observed the highest ratio of heterozygous genotypes to homozygous genotypes (Het/Hom) in the SSIP samples among all Asians, and the levels present in the SSIP were comparable to those in Europeans (Figure S10). The trend in the distribution of Het/Hom remained consistent even when adjusted for sample size and sequencing coverage (Figures S10, S11), where Africans generally displayed Het/Hom of around 2, East Asians around 1.4, and South Asians (SSIP, GIH from Complete Genomics) and Europeans around 1.6.

### Mitochondria and Y chromosome haplogroup membership

We assigned the 36 SSIP mitochondria (mtDNA) sequences to known mitochondria haplogroups with HaploGrep [19]. The majority of the samples were assigned to the haplogroups M, N and R (Table 2) which were found in high frequencies in South Asian populations [20], [21], [22]. However, the remaining 9 samples were assigned to haplogroups F, HV, T, U and W. Other than haplogroup F that was found mostly in East Asia, the other four haplogroups were predominantly present in European populations. In a similar analysis of chromosome Y for the 11 male SSIP samples (Table 3), eight of the Y chromosomes were assigned to haplogroups that were predominantly found in South Asia (F: primarily a south Indian Dravidian-speaking haplogroup; H: present mainly in the Indian subcontinent; L: present mainly in South and Central Asia). Two samples were assigned to haplogroups J2b2 that were present mainly in the Mediterranean and Southern Europe, while the remaining sample was assigned to R1a1, a haplogroup that is mostly present in Europe but is found at low frequencies in South Asia.

**Table 2:** Mitochondria haplogroup assignment for the 36 SSIP samples.

Sample	Haplogroup	Sample	Haplogroup	Sample	Haplogroup
SSI003	M30d	SSI017	T2a1a	SSI029	R6
SSI004	F1c1a	SSI018	M40	SSI030	HV12b
SSI005	M33a+146	SSI019	M7c3c	SSI031	M3a1
SSI006	M	SSI020	M34	SSI032	U1a3
SSI008	N8	SSI021	M6a1	SSI033	U1a3
SSI009	M53	SSI022	M36	SSI034	R8a1a1
SSI010	M36d1	SSI023	W3a1	SSI035	R6a
SSI011	M66	SSI024	HV12b	SSI036	M5c1
SSI012	M2a'b	SSI025	U7	SSI037	M40a
SSI013	HV14	SSI026	M35a1	SSI038	M5a2a
SSI014	M3a1	SSI027	M34	SSI039	M66
SSI015	M3a2	SSI028	M35a1	SSI040	M6a1

**Table 3:** Chromosome Y haplogroup assignment for the 11 SSIP male samples.

Sample	Haplogroup (trunk)	Haplogroup (branch)	Haplogroup (branch)
SSI004	J	J2b2	J2b2
SSI006	L	L1	L1
SSI009	H	H1	H1
SSI012	L	L1	L1
SSI014	L	L1	L1
SSI023	F	F	F
SSI029	H	H1	H1
SSI031	J	J2b2	J2b2
SSI032	R	R1a1	R1a1
SSI034	H	H1	H1
SSI036	L	L1	L1

To investigate the correlation between the admixture analysis and the haplogroup assignments, we stratified the 36 samples according to the mitochondria haplogroups into a European dominant group (haplogroups HV, T, U and W; 8 samples) and a non-European dominant group (haplogroups F, M, N and R; 28 samples) and compared the estimated degree of European admixture between these two groups when we assumed six distinct populations in the admixture analysis (Table S8). We assumed six distinct populations as this yielded amongst the lowest cross-validation errors and was the most parsimonious choice (Figure S15). We observed a significant elevation of the European component (9.1% versus 6.5%, *P* = 0.047) in the samples identified with European-dominant mitochondrial haplogroup assignment, despite this not being evident in the PCA with autosomal SNPs (Figure S12). We observed that the extent of European admixture in the SSIP was about 7% (at *K* = 6, Figure S15), which was lower than previous studies where two reported around 50% north Indian ancestry in 17 Indian subgroups [11], [23]. However, the results between the different studies are not directly comparable due to the makeup of the European populations, since our analyses have considered five European subgroups (CEU, FIN, GBR, IBS, TSI) while the published reports have used only CEU to represent Europeans in the admixture estimation. This can be seen in our admixture analysis that assumed three distinct populations (*K* = 3), where the SSIP is observed to possess an average of 53% European admixture (Figure S16), which is in fact in agreement with previous reports.

### Admixture with ancient genomes

By randomly choosing one sample from each of the 16 populations, we calculated the *Dstatistic* metric to investigate the relative extent of admixture of two ancient hominid genomes, a Neanderthal and a Denisovan, into modern humans. As *Dstatistic* required the input of four genomes, the analysis was anchored with a specific SSIP sample (SSI033 as G1) together with an ancient hominid and the chimpanzee genome, and we varied only the sample that was used in this four-sample analysis (Table 4). We observed significantly greater extent of Neanderthal genome in East Asians (CHB, JPT) relative to SSIP, but lesser in the Luhya Kenyans (LWK) than SSIP. This was consistent with the estimations by Wall and colleagues, despite the use of Gujarati Indians as their South Asian samples [24]. There was also evidence to suggest a greater degree of Denisovan admixture in northern Han Chinese (CHB) than SSIP, although this observation was not reproduced for the other two East Asian populations (CHS, JPT). To evaluate the robustness of these analyses to the choice of samples and sequence coverage, we additionally performed the *Dstatistic* calculation on five randomly chosen pair of samples from each population (anchored with a different SSIP sample as G1), and also performed the same analyses on the populations sequenced by Complete Genomics (to a minimum coverage of 51X). These additional analyses indicated that our original observations were robust (Table 5, Table S9).

**Table 4:** Analysis of admixture with ancient hominid genomes, anchored with one SSIP genome (SSI033 as G1 in Dstatistic) and the chimpanzee genome.

Neanderthal						Denisovan					
Sample (G2)	*N_BABA_*	*N_ABBA_*	%*Dstat*	%SE	Z	Sample (G2)	*N_BABA_*	*N_ABBA_*	%*Dstat*	%SE	Z
**Americans**											
NA19720_MXL	61,869	62,711	−0.676	0.936	0.543	NA19749_MXL	56,423	56,824	−0.35	0.74	1.24
HG01271_CLM	62,637	63,033	−0.315	0.988	0.505	HG01342_CLM	59,852	60,988	−0.94	0.71	1.93
HG01060_PUR	63,134	63,171	−0.029	0.959	−0.894	HG01191_PUR	56,526	57,331	−0.71	0.68	1.50
**Africans**											
NA19908_ASW	72,284	69,292	2.113	0.757	−1.275	NA20299_ASW	58,889	61,285	−1.99	0.68	1.48
NA19401_LWK	73,400	70,380	2.100	0.765	−2.311	NA19327_LWK	65,428	68,000	−1.93	0.64	1.34
NA19236_YRI	73,774	69,606	2.907	0.729	−1.926	NA19160_YRI	66,408	67,672	−0.94	0.64	1.36
**Asians**											
NA18978_JPT	61,398	62,449	−0.849	0.935	−2.548	NA19078_JPT	56,313	55,554	0.68	0.67	−0.75
NA18645_CHB	61,203	62,837	−1.317	0.945	−3.014	NA18577_CHB	55,720	56,822	−0.98	0.75	2.06
HG00500_CHS	61,443	63,314	−1.500	0.899	−1.894	HG00543_CHS	55,647	56,643	−0.89	0.74	1.16
SSM097	57,291	60,031	−2.335	0.888	1.815	SSM059	52,166	52,617	−0.43	0.71	1.37
**Europeans**											
NA20755_TSI	61,990	63,008	−0.814	1.053	−1.291	NA20813_TSI	56,229	56,837	−0.54	0.77	1.74
NA07056_CEU	62,356	62,641	−0.228	1.001	−1.123	NA12775_CEU	56,260	55,649	0.55	0.68	1.30
HG00315_FIN	62,523	62,366	0.126	0.991	−1.195	HG00275_FIN	56,541	56,308	0.21	0.73	1.87
HG01624_IBS	62,210	62,496	−0.229	0.952	−0.994	HG01620_IBS	56,162	56,823	−0.59	0.68	−0.49
HG00261_GBR	62,198	61,291	0.734	0.879	−1.447	HG00263_GBR	56,652	55,975	0.60	0.76	1.78

**Table 5:** Dstatistic analysis with ancient genomes for 5 randomly selected paired samples from each population from 1KGP and SSMP, anchored with a different SSIP sample (G1) and the chimpanzee genome in each of the 5 iterations.

Population	Neanderthal	Denisovan
G2	Mean %Dstatisitc	Standard deviation	Mean %Dstatisitc	Standard deviation
**Americans**				
MXL	−0.8287	0.51	−0.3476	0.38
CLM	−0.3085	0.16	−0.9630	0.70
PUR	−0.1668	0.17	−0.7225	0.55
**Africans**				
ASW	2.1022	0.82	−1.8012	0.45
LWK	2.5654	0.29	−1.6356	0.47
YRI	2.9993	0.31	−0.9381	0.12
**Asians**				
JPT	−0.8431	0.48	0.7085	0.67
CHB	−1.0029	0.69	−0.9975	0.81
CHS	−1.1559	0.58	−0.8884	0.80
MAS	−1.4695	0.67	−0.4289	0.24
**Europeans**				
TSI	−0.7051	0.21	−0.5580	0.51
CEU	−0.2317	0.08	0.5491	0.47
FIN	−0.2016	0.22	0.2371	0.24
IBS	−0.2121	0.22	−0.5146	0.42
GBR	0.7361	0.62	0.6602	0.64

## Discussion

The Singapore Sequencing Indian Project has produced a publicly available genomic resource by sequencing the genomes of 36 South Asian Indians from Singapore at a target coverage of 30X. This complements the existing resource of 96 Southeast Asian Malays from the SSMP, as well as 1,092 samples from Phase 1 of the 1000 Genomes Project. By comparing against a panel of 132 South Asians with well-defined geographical origins, the 36 Singapore Indians were found to be genetically closer to the South Asians from south India. The assignment of Y chromosomes and mitochondria to known haplogroups identified 18% and 22% of the samples respectively to belong to haplogroups that are predominantly present in Europeans, and these memberships were not discernible in the PCA using autosomal SNPs of SSIP with other global populations, even though there were marginally higher degree of Caucasian admixture in these samples that were statistically significant.

The PCA of the South Asian samples revealed greater genetic heterogeneity in Asian Indians that originated from northern parts of India, while those that originated from the south were considerably more homogeneous (see Figure 2C). In an independent analysis of HapMap Gujarati Indians with Asian Indians from the Singapore Genome Variation Project, around a third of the Gujarati Indians were found to cluster with the Singapore Indians (Figure S13). With an original sample size of 38, the SSIP is hardly representative of the complex genomic diversity that is present in the Indian subcontinent, nor does it contain geographically well-distributed samples to yield deeper insights into the migratory history of South Asians. Many theories have been proposed on the differentiation between Indo-Aryan and Dravidian-speaking Indians, and we emphasize the SSIP is not intended to investigate the different hypotheses, although it can serve as a potential population panel when more South Asian genetics data become available. Individuals in the SSIP have been sampled from Singapore, a location which is geographically distant from India, and migration and inter-marriages likely will have confounded genetic membership to specific geographical origins in south India, and thus the SSIP is likely to be more representative of cosmopolitan Indians in Singapore.

While our assessment of intra-population diversity considers the extent of SNP sharing between pairs of samples in a population, the variance of this metric provides an effective measure of the genetic homogeneity of population labels. For example, the admixed Mexican population exhibited the largest spread in the pairwise distances, even though the median distance for Mexicans was in the same range as with the Caucasians (see Figure 5). This suggests that for subjects labeled as Mexicans, there are pairs of samples where the extent of SNP sharing was as similar as East Asians, while at the same time there are pairs within the population that were significantly more diverse than between pairs of SSIP samples. The downstream implication to such variable degree of intra-population diversity is the interpretation and relevance of population labels as surrogate for genomic information in the practice of public health. An example of this is in warfarin pharmacology. Although polymorphisms in *VKORC1* and *CYP2C9* can explain up to 70% of dosage variance between populations [25], it is still common to rely on race or ethnic labels to identify the loading dosages when initiating warfarin therapy in the absence of information from genetic screening [25], [26]. A population with a large variance for the intra-population diversity suggests that the adherence to self-reported population labels may serve as a poor surrogate for the underlying pharmacogenomics, which was evident in the larger range of warfarin international normalized ratio (INR) for African Americans and Caucasians, as compared to the Chinese and Japanese (see figure 1 of [26]).

The SSIP resource is expected to be a timely complement to Phase 3 of the 1 KGP, which has sequenced the genomes of samples from three additional migrant South Asian groups in Houston (Gujarati Indians) and the United Kingdom (Sri Lankan Tamils, Indian Telegus), as well as two native South Asian groups in Bangladesh (Bengali) and Pakistan (Punjabi). Similar to the Phase 1 design, these five groups have been sequenced at a low coverage of between 2- to 6-fold, and it can be useful to evaluate whether the availability of the SSIP data will benefit the process of variant calling for these populations that have undergone low-pass sequencing. The variants for the 36 SSIP samples are publicly available in the variant call format (VCF), and these can be accessed along with phased haplotypes for the SSIP samples at http://www.statgen.nus.edu.sg/~SSIP.

## Materials and Methods

### Samples

Subjects enrolled in the SSIP consisted of 38 subjects, 12 males and 26 females, from the Multi-Ethnic Cohort (MEC) of the Singapore Population Health Study who self-reported themselves as Singapore Indians. This is a cross-sectional survey on individuals of ages between 40 and 65 years old, the exclusion criteria at the time of the initial enrolment into the MEC were: (i) below 21 years of age; (ii) having any mental condition that may interfere with the participant's competency in giving informed consent; and were suffering from any of the following conditions (iii) renal failure; (iv) stroke; (v) cancer; (vi) heart disease (including congenital conditions). We confirmed the ethnic membership of each individual via a telephone survey to verify that both sets of grandparents similarly self-reported to be South Asian Indians. Informed consent was obtained from all participants and ethical approvals were obtained for the Singapore Population Health Study and the extension to perform whole-genome sequencing from two independent Institutional Review Boards at the National University Hospital (Singapore) and the National University of Singapore respectively.

### Sample preparation and sequence data generation

The blood samples of all 38 Singapore Indians were extracted from the Singapore BioBank, and DNA extraction was performed at the Defence Medical and Enviromental Research Institute according the protocol by Illumina, with DNA quantification performed using picogreen and the SpectraMax Gemini EM microplate reader (with spectrophometic set at 480/520 nm) to ensure DNA concentration for each sample was at least 50 ng/µl. Whole genome sequencing was performed at the Illumina facility at Hayward, California, USA, using the Illumina HiSeq 2000 sequencer, where each sample was run on a unique lane to achieve a target coverage of 30-fold with 2×100 paired-end reads and a target insert size of between 300–400 bp. To ensure consistent and quality sequencing, multiple quality control procedures were adopted: (i) in preparing the libraries, the Bioanalyzer was used to ensure DNA quality and size distribution; (ii) a short paired-end sequencing reaction was applied to each sample after library preparation to ensure the extent of GC bias and the observed sequencing quality were within normal ranges; (iii) sequencing of each sample was performed on a unique lane, with the condition that at least 80% of the generated bases must attain a quality score of at least 30, failing which the sample was re-sequenced. Each sample was also genotyped on the Illumina Omni2.5 M microarray, where genotype calling was performed with the proprietary GenomeStudio software by Illumina.

### Read assembly and alignment

Assembly and alignment of each individual genome to the human reference genome (National Center for Biotechnology Information, NCBI build 37) was performed using the proprietary Illumina CASAVA version 1.9.0a1_110909 assembler. CASAVA aligned sequence reads using Eland v2e, and the aligned reads for each sample were then consolidated into the BAM format file [27].

### Variant discovery

Two methods were used to call SNPs and indels: (i) single sample calling by CASAVA; and (ii) multi sample calling by GATK version 2.1.8 [28], [29] (see Supplementary Methods for details). We assessed the performance of both methods by comparing the concordance of the genotypes called by CASAVA and GATK with the genotypes reported in the Illumina Omni2.5 M array. We reported only variants that were identified by both CASAVA and GATK, but used the genotype calls from the software that yielded a higher concordance rate. SNP annotation was performed using SNPEff version 3.1 b [17], while the functional impact of the SNPs were predicted using both SIFT [30] and Polyphen [31] where a non-synonymous SNP was defined as damaging if SIFT yielded a score ≤0.05 and PolyPhen-2 yielded a score ≥0.95. We defined LOF SNPs as those that were annotated by SNPEff to be nonsense mutations, splice-site mutations, or frame-shifts caused by indels [2], and the Database for Annotation, DAVID [32] was used to identify biological pathways that were enriched with LOF SNPs in our samples. In addition, we mapped the LOF variants in our samples to the COSMIC [33], [34] and previous discoveries from GWAS [35] with the online SNPnexus platform [36] to identify any functional impact of these LOF SNPs. SVs were called with 4 methods: (i) BreakDancer v1.1._2011_02_21 [37]; (ii) VariationHunter Release_v0.3 [38]; (iii) Pindel version 0.2.2 [39]; and (iv) Delly v0.0.5 [40], although the focus was primarily on deletions. Large deletions of size 50 bp to 10 Mbp that were successfully called by at least one of the four algorithms were consolidated, whereby for structural deletions that were detected by multiple methods, the boundaries were obtained by considering the union of the deleted regions from these methods (see Supplementary Methods). A SNP or indel is defined to be novel if it is not present in dbSNP 137 or the SSMP [3], while a structural deletion is defined to be novel if there is less than 50% overlap with previously reported deletions in the SSMP, 1 KGP and DGV released 2013-07-23 [41].

### Assessing population structure

PCA was used to assess the population structure of the SSIP samples with samples from worldwide populations using the *pca* option in the software *eigenstrat* [42]. As part of the sample QC process, a PCA was performed with 420,817 autosomal SNPs on the 38 SSIP samples and 268 samples from the SGVP [43] to ensure that the SSIP samples clustered together with the SGVP Indian samples. The SSIP samples that remained after QC were jointly analyzed with 96 samples from the SSMP and 1,092 samples from 14 populations in Phase 1 of 1 KGP in a PCA of the 16 populations on 217,302 SNPs (Table S1). A third PCA was performed with 202,600 SNPs on the SSIP samples with 132 South Asian samples from 25 well-defined Indian groups [44], where the latter samples can be broadly categorized into Southern Indians and Northern Indians according to the latitude of the sampling location (Table S2). To estimate the membership of each sample in the 16 populations to dominant population groups in the world, an admixture analysis was performed using ADMIXTURE version 1.22 program [45] with 6,519,079 autosomal SNPs, where the number of ancestral population (*K*) was set to range from 2 to 16.

### Assessing population diversity

We measured the genetic diversity of SSIP, SSMP and 14 populations in Phase 1 of 1 KGP with a distance metric calculated between every possible pair of samples in each of the 16 populations, defined as 
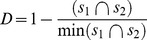
, where min(*S*_1_,*S*_2_) is the minimun number of SNPs in two samples denoted as *S*_1_ and *S*_2_, and 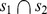 represents the number of SNPs present in both *S*_1_ and *S*_2_. A high *D* value thus reflects a lower degree of SNP sharing, or greater genetic heterogeneity, between the two samples; a low *D* value indicates that SNPs present in *S*_1_ are likely to be present in *S*_2_, thus reflecting greater genetic homogeneity between the two samples. To evaluate whether the number of samples in each population influenced the comparison, we additionally performed the analysis with the same number of samples selected randomly from each population. To ensure that the results of this analysis were robust to sequencing coverage and the method used for variant calling, we also measured *D* for samples from six populations in 1 KGP (CEU, CHB, JPT, LWK, TSI and YRI) that were sequenced by Complete Genomics [46] at a higher coverage of 51-fold to 89-fold (see Table S1). For each individual, we also measured the ratio of heterozygous genotypes to homozygous genotypes (Het/Hom) across all the autosomal SNPs. A population-level metric is calculated as the average of the Het/Hom across all the individuals in a specific population. This metric was similarly calculated with the same number of samples chosen randomly from each population, and including samples from Complete Genomics to calibrate against sequencing coverage and variant calling differences.

### Mitochondria and chromosome Y haplogroup assignment

Every SSIP individual was assigned to a unique mitochondria haplogroup on the basis of the complete sequence of the mtDNA. This was performed with haplogrep with reference to PhyloTree mtDNA tree Build 15 [19], [45] using a FASTA-based input generated with the consensus calls from GATK and *mpileup* in SAMTOOLS [27] (see Supplementary Methods for details). Each of the 11 male subjects in SSIP was also assigned to a unique chromosome Y haplogroup defined using a maximum likelihood approach against the 2008 chromosome Y tree with Yfitter [47]. An input VCF file of the detected variants was generated with *mpileup* in SAMTOOLS, which was subsequently converted to the qcall input file used by Yfitter for haplogroup assignment. Default settings for Yfitter and haplogrep were used in our analyses.

### Relatedness with ancient genomes

We evaluated the relative degree of relatedness between one SSIP sample (SSI033, chosen randomly) and a randomly chosen sample from each of the 15 populations (SSMP, 1 KGP) with an ancient genome using the *Dstatistic* metric [48]. Calculating this metric requires information at bi-allelic transversion substitutions as transition mutations are likely results of deamination of cytosine residues in ancient DNA [49] (denoted generically as alleles *A* and *B*) sites from two modern human genomes (*G*_1_, *G*_2_), an ancient genome (either Denisovan or Neanderthal) and the chimpanzee genome, and 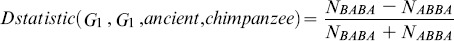 where *N_BABA_* denote the total number of sites where *G*_1_ and the ancient genome carried allele *B* while *G*_2_ and the chimpanzee genome carried allele *A*; and *N_ABBA_* denote the total number of sites where *G*_1_ and the chimpanzee genome carried allele *A* while *G*_2_ and the ancient genome carried allele *B*. At sites where the ancient genome carries different alleles to the chimpanzee genome, *Dstatistic* thus measures the deviation in similarity between the ancient genome and each of the two modern genomes. The genome was divided into *M* non-overlapping blocks of 5 Mb and a jack-knife approach was used to calculate the mean, standard error and Z score of *Dstatistic*. Two ancient genomes were considered: (i) the Denisovan genome, sequenced on the Illumina Genome Analyzer IIx at a coverage of 30-fold [50]; and (ii) the Neanderthal genome, sequenced on the Illumina HiSeq at a coverage of 50-fold (http://www.eva.mpg.de/neandertal/index.html).

A full description of the Methods can be found in the Text S1 and Supplementary Methods at the SSIP website.

## Supporting Information

Figure S1 Mean and standard deviation of insert sizes for each sample. The vertical blue bars represent mean insert sizes while the red line shows the standard deviation of insert sizes from the paired-end sequencing reads. No outliers were found.
(TIF)

Figure S2 Total number of reads for each sample. Total number of reads sequenced for all samples fall within an acceptable range. Blue bars represent reads that passed QC while red bars represent reads that failed QC.
(TIF)

Figure S3 Total paired reads for each sample. Vertical blue bars represent reads that passed QC while red bars represent reads that failed QC. No unusual trend observed.
(TIF)

Figure S4 Read depth summary statistics for each sample. Sample SSI007 displayed a median read depth less than the targeted depth of 30X (red line) and is subsequently excluded from downstream analyses. 75^th^ and 25^th^ percentiles are represented by green line and blue line respectively.
(TIF)

Figure S5 Principal Component Analysis (PCA) of samples from Singapore Sequencing Indians Project (SSIP) and Singapore Genome Variation Project (SGVP). A set of 420,817 SNPs common between the 38 samples from SSIP (blue circles) and 268 samples from the SGVP, which includes 96 Chinese (red), 89 Malays (green) and 83 Indians (sky blue) were used to generate a PCA plot. The analysis revealed one sample from SSIP (SSI016) to be of closer proximity to Malays (SGVP_MAS), this sample was removed from downstream analysis.
(TIF)

Figure S6 Genotype concordance rate for autosomes SNPs. Comparison of genotype concordance rate between CASAVA (blue) and GATK (red) SNPs calling with reference to Omni 2.5 M array for autosomal SNPs. Chromosome number is displayed at horizontal axis. CASAVA outperformed GATK across all chromosomes.
(TIF)

Figure S7 Pairwise populations FST between SSIP and other 15 populations on bi-allelic SNPs of autosomal chromosomes. Blue bar is the mean pairwise population FST for SSIP and another population for common SNPs between the two populations, green bar represents mean pairwise population FST for SSIP and another population for common SNPs across entire population panel (total of 4,460,176 SNPs for original sample sizes in (A), 4,360,323 for 36 samples each population in (B)). Red circle shows number of common SNPs between a pair of populations that was used for mean FST calculation.
(TIF)

Figure S8 Genetic diversity measured by distance metric. Intra population diversity measured for all possible pairs of sample in each population for (A) original sample size; (B) normalized sample size by randomly selecting 36 samples from each population. IBS was removed from the analysis because its sample size was less than 36 samples. We do not observe any deviation between original samples size and normalized samples size and thus this analysis is not sensitive to sample size variation.
(TIF)

Figure S9 Intra population diversity for 7 populations in 1 KGP and Complete Genomics. Intra population diversity base on distance measure *D* for 7 populations for (A) 1 KGP (average coverage of 5X) and (B) Complete Genomics (deep coverage of 51-89X). Label at axis X show information of continent, population, sample size and total number of pair. Identical trend was observed regardless of low or deep sequencing coverage. African populations have the highest intra-population diversity score while Asian populations have the lowest, GIH (Northern Indians) and SSIP are slightly above Europeans.
(TIF)

Figure S10 Heterozygous to homozygous ratio. (A) Boxplot of original sample sizes heterozygous to homozygous ratio in each population. (B) We randomly selected 36 samples from each population (SSIP, SSMP and 1 KGP) to calculate single sample heterozygous to homozygous ratio. SSIP has the highest ratio than all other Asian populations, indicating SSIP is more diverse than East Asian populations (JPT, CHB, CHS) and Southeast Asian population (SSMP).
(TIF)

Figure S11 Heterozygous to homozygous ratio for Complete Genomics samples. Heterozygous to homozygous ratio for samples from Complete Genomics color coded by continent, red represents Africans, green for Asians while skyblue for Europeans. Het/Hom ratio obtained in deep sequencing samples (Complete Genomics) and shallow sequencing samples (1 KGP) are within the same ranges for all compatible populations. Axis X show information of sample id and population.
(TIF)

Figure S12 Principal component analysis of 16 world populations. PCA of 1,224 samples from SSIP, SSMP and 14 populations from Phase 1 of the 1 KGP, where the samples are grouped and color-coded by continents (legend). Blue circles are 26 SSIP samples and the remaining 8 SSIP individuals (yellow circles) are with European dominant mitochondria haplogroup assignment.
(TIF)

Figure S13 Principal component analysis of 83 Indians from Singapore Genome Variation Project and 85 Gujarathi from Hapmap 3. PCA on a set of 30,927 SNPs for 83 Singapore Indians (blue) from SGVP and 85 Gujarati Indians in Houston (skyblue) from Hapmap 3.
(TIF)

Figure S14 Admixture analysis on 104 samples from 20 Indian subgroups [11] and 36 SSIP individuals. An analysis of admixture on the 20 Indian subgroups and SSIP with ADMIXTURE program, *K* is the number of distinct populations that varied between 2 and 5. Black windows highlights the position of the Chencu, Austro-Asiatic groups (Kharia and Santhal), Northern Indians and Southern Indians (see Table S2 for the categorization of the 20 Indian subgroups into Southern or Northern Indians).
(TIF)

Figure S15 Cross validation error for admixture analysis with *K* from 2 to 15 performed on 16 populations. Cross-validation error in the admixture analysis at different values of ancestral groups (*K*), which we have allowed to range from 2 to 15. It was observed that while *K* = 7 yielded the lowest cross-validation error, *K* = 6 yielded a difference that was less than 0.01 and thus *K* = 6 was chosen for reporting in the main text on the basis of parsimony.
(TIF)

Figure S16 European component in 36 SSIP individuals from admixture analysis. Bars represent average of European component in 36 SSIP samples from the admixture analysis on 6,519,079 SNPs of 16 populations in which 14 populations from 1 KGP (Table S1), SSMP and SSIP. *K* indicates the number of ancestral populations. At *K* = 3, the three ancestral populations are African, European and Asian (Figure 3B) where an average of 53.42% European component was found in 36 SSIP individuals. When *K* increased, more ancestral populations contributed to the entire admixture panel that likely to dilute the European component possessed by SSIP individuals.
(TIF)

Table S1 Description of populations used for comparison with SSIP.
(DOC)

Table S2 Description of 25 Indian groups extracted from Reich et al. 2009.
(DOC)

Table S3 Transition to transversion ratio (Ts/Tv). (A) Ts/Tv for bi-allelic SNPs, (B) Ts/Tv after the removal of CpG exonic transition SNPs.
(DOC)

Table S4 Summary of single sample Loss-of-function (LOF) variants.
(DOC)

Table S5 List of pathways affected by Loss-of-function (LOF) variants.
(DOC)

Table S6 List of Loss-of-function (LOF) variants found in COSMIC database.
(DOC)

Table S7 List of Loss-of-function (LOF) variants related to GWAS studies.
(DOC)

Table S8 The proportions for 6 ancestral populations extracted from the output of ADMIXTURE program.
(DOC)

Table S9 D statistic analysis with ancient genome for 5 randomly selected pairwise samples, anchored with different SSIP sample (G1). (A) Neanderthal as ancient hominid, (B) Denisovan as ancient hominid.
(DOC)

Text S1 Supplementary methods.
(DOC)
