# Supplementary figures and images for: Insights into the Genetic Structure and Diversity of 38 South Asian Indians from Deep Whole-Genome Sequencing
Source: PLoS Genet. 2014 May 15;10(5):e1004377. doi: 10.1371/journal.pgen.1004377 (PMC4022468; doi:10.1371/journal.pgen.1004377)

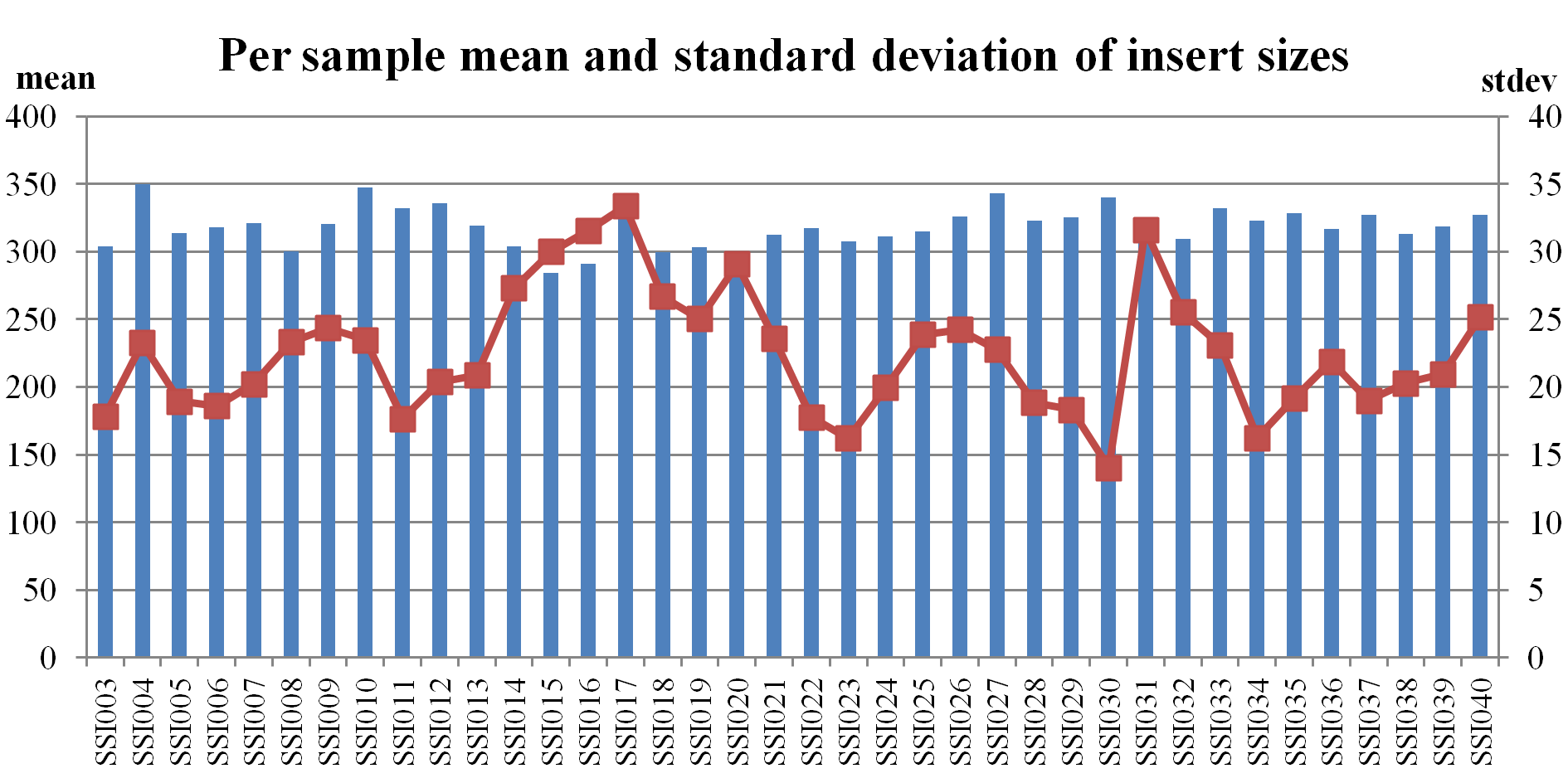

Supplement: Figure S1 — Mean and standard deviation of insert sizes for each sample. The vertical blue bars represent mean insert sizes while the red line shows the standard deviation of insert sizes from the paired-end sequencing reads. No outliers were found. (TIF) [file pgen.1004377.s001.tif]

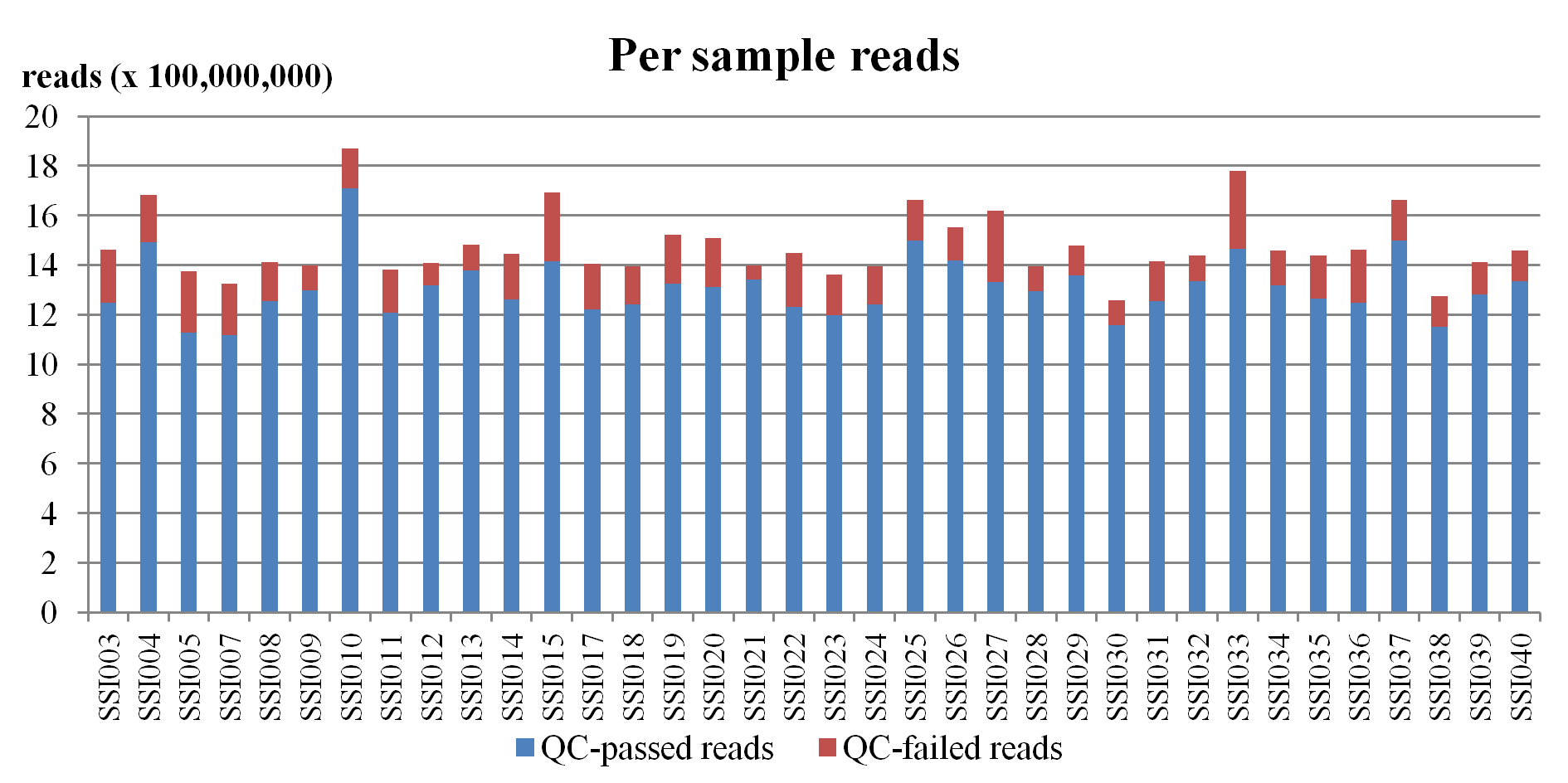

Supplement: Figure S2 — Total number of reads for each sample. Total number of reads sequenced for all samples fall within an acceptable range. Blue bars represent reads that passed QC while red bars represent reads that failed QC. (TIF) [file pgen.1004377.s002.tif]

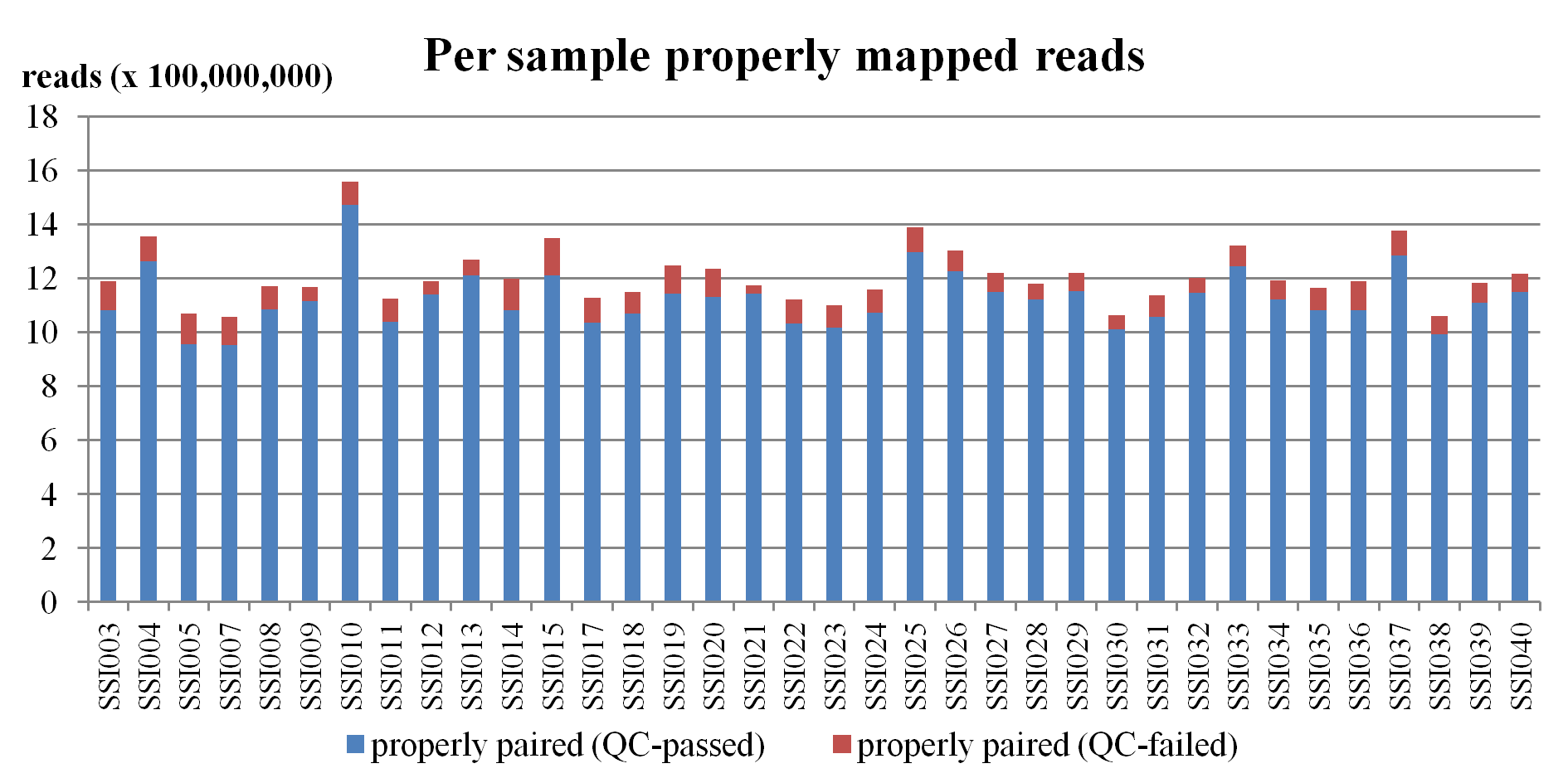

Supplement: Figure S3 — Total paired reads for each sample. Vertical blue bars represent reads that passed QC while red bars represent reads that failed QC. No unusual trend observed. (TIF) [file pgen.1004377.s003.tif]

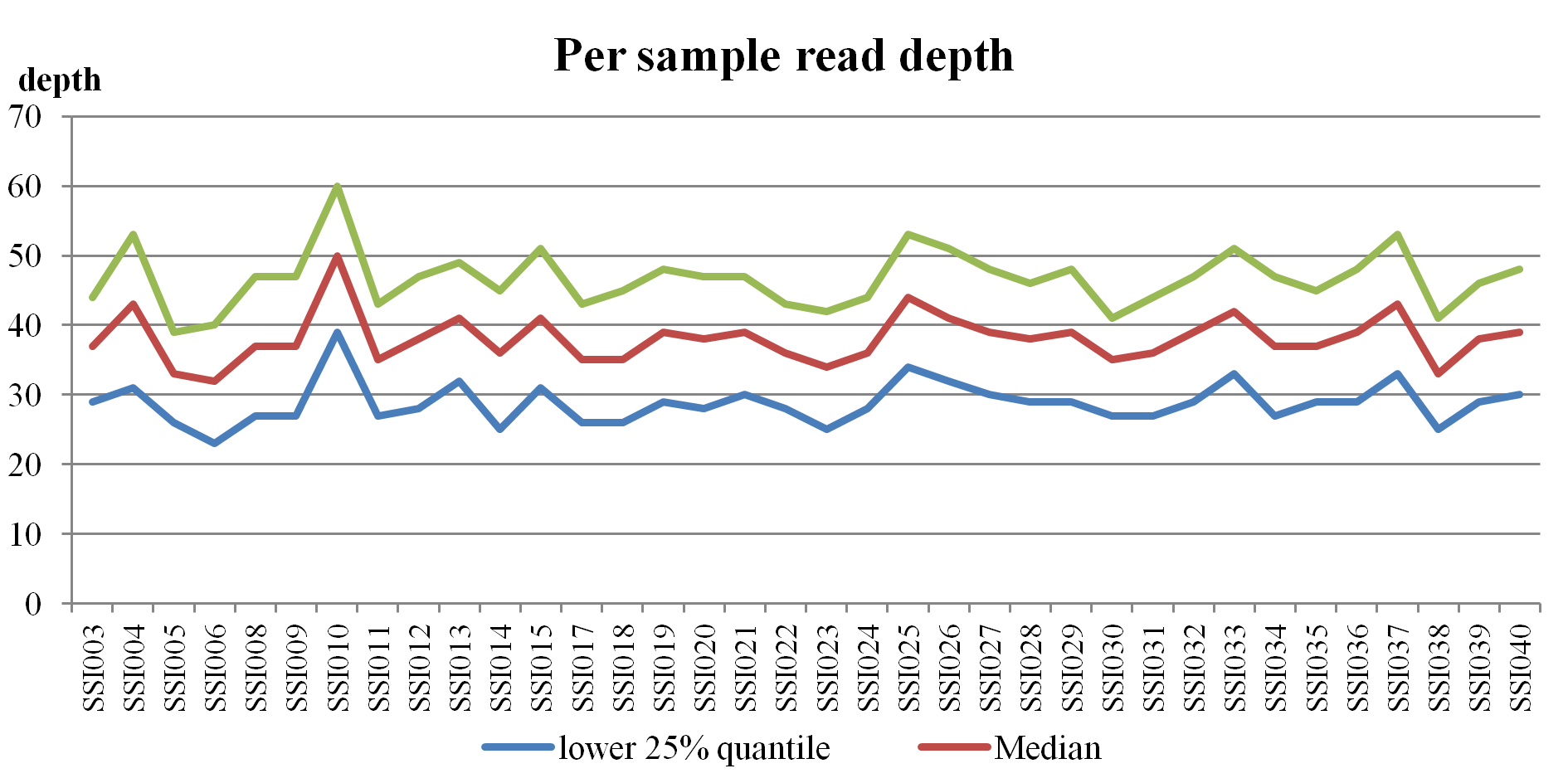

Supplement: Figure S4 — Read depth summary statistics for each sample. Sample SSI007 displayed a median read depth less than the targeted depth of 30X (red line) and is subsequently excluded from downstream analyses. 75th and 25th percentiles are represented by green line and blue line respectively. (TIF) [file pgen.1004377.s004.tif]

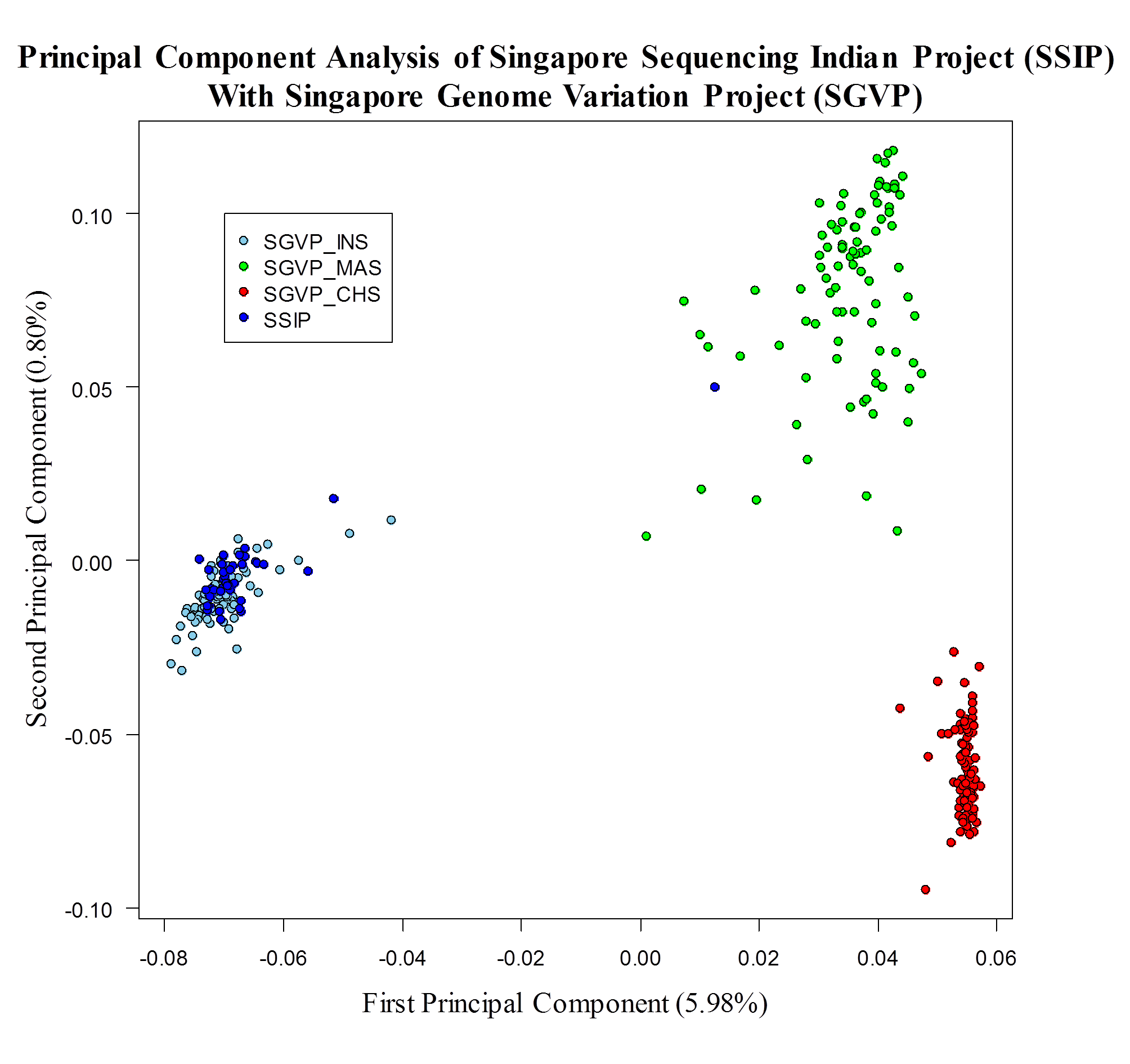

Supplement: Figure S5 — Principal Component Analysis (PCA) of samples from Singapore Sequencing Indians Project (SSIP) and Singapore Genome Variation Project (SGVP). A set of 420,817 SNPs common between the 38 samples from SSIP (blue circles) and 268 samples from the SGVP, which includes 96 Chinese (red), 89 Malays (green) and 83 Indians (sky blue) were used to generate a PCA plot. The analysis revealed one sample from SSIP (SSI016) to be of closer proximity to Malays (SGVP_MAS), this sample was removed from downstream analysis. (TIF) [file pgen.1004377.s005.tif]

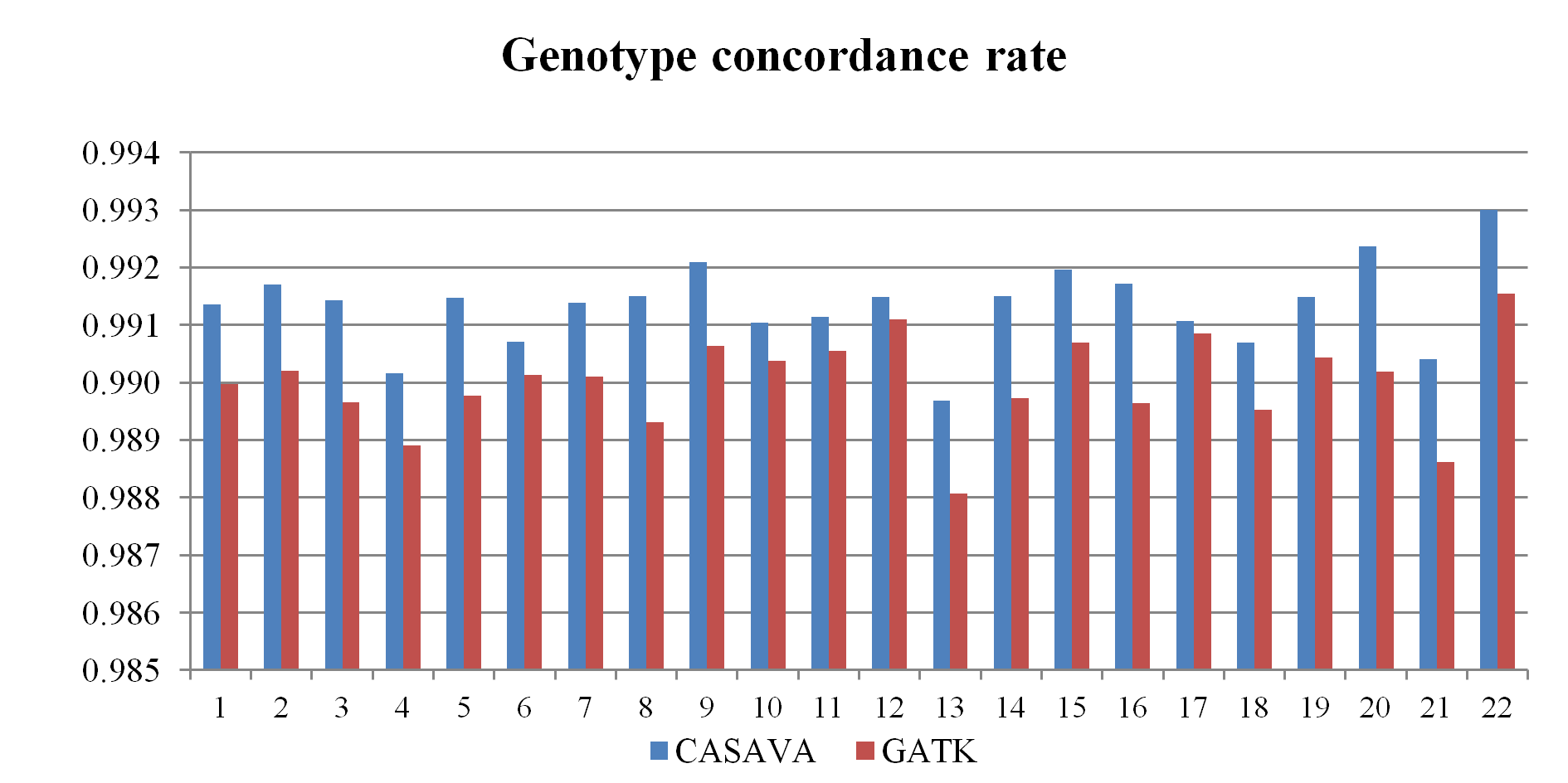

Supplement: Figure S6 — Genotype concordance rate for autosomes SNPs. Comparison of genotype concordance rate between CASAVA (blue) and GATK (red) SNPs calling with reference to Omni 2.5 M array for autosomal SNPs. Chromosome number is displayed at horizontal axis. CASAVA outperformed GATK across all chromosomes. (TIF) [file pgen.1004377.s006.tif]

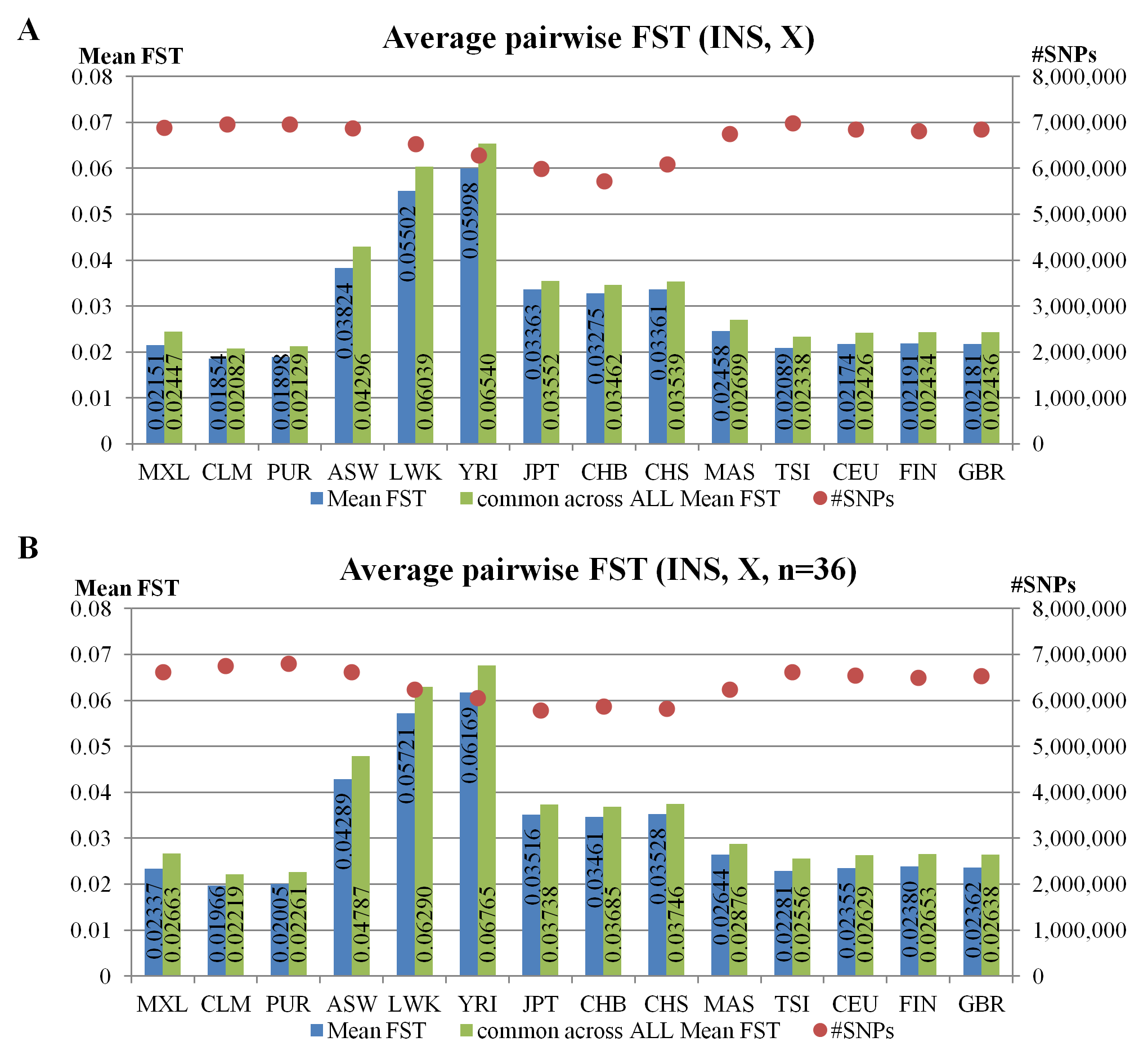

Supplement: Figure S7 — Pairwise populations FST between SSIP and other 15 populations on bi-allelic SNPs of autosomal chromosomes. Blue bar is the mean pairwise population FST for SSIP and another population for common SNPs between the two populations, green bar represents mean pairwise population FST for SSIP and another population for common SNPs across entire population panel (total of 4,460,176 SNPs for original sample sizes in (A), 4,360,323 for 36 samples each population in (B)). Red circle shows number of common SNPs between a pair of populations that was used for mean FST calculation. (TIF) [file pgen.1004377.s007.tif]

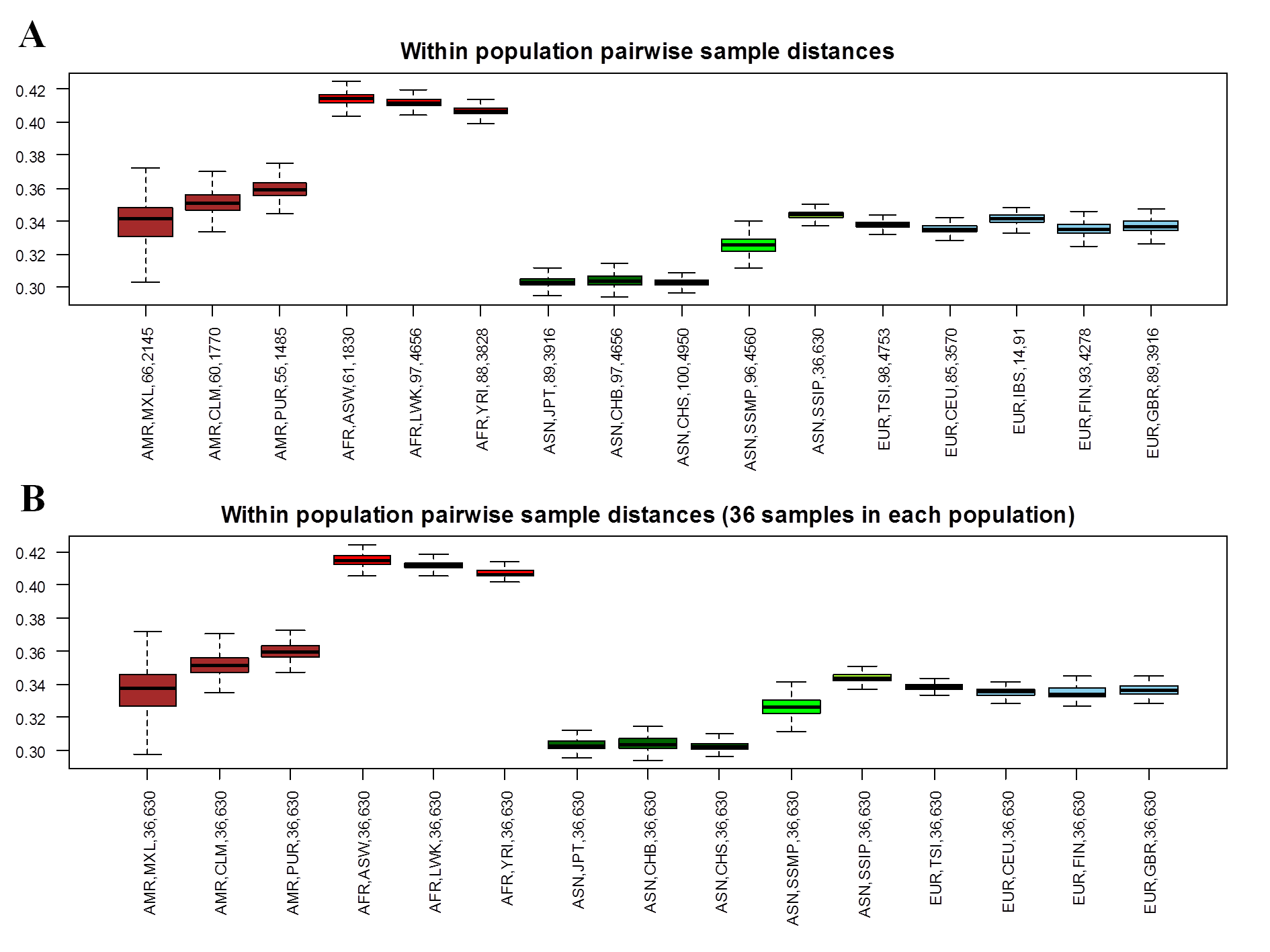

Supplement: Figure S8 — Genetic diversity measured by distance metric. Intra population diversity measured for all possible pairs of sample in each population for (A) original sample size; (B) normalized sample size by randomly selecting 36 samples from each population. IBS was removed from the analysis because its sample size was less than 36 samples. We do not observe any deviation between original samples size and normalized samples size and thus this analysis is not sensitive to sample size variation. (TIF) [file pgen.1004377.s008.tif]

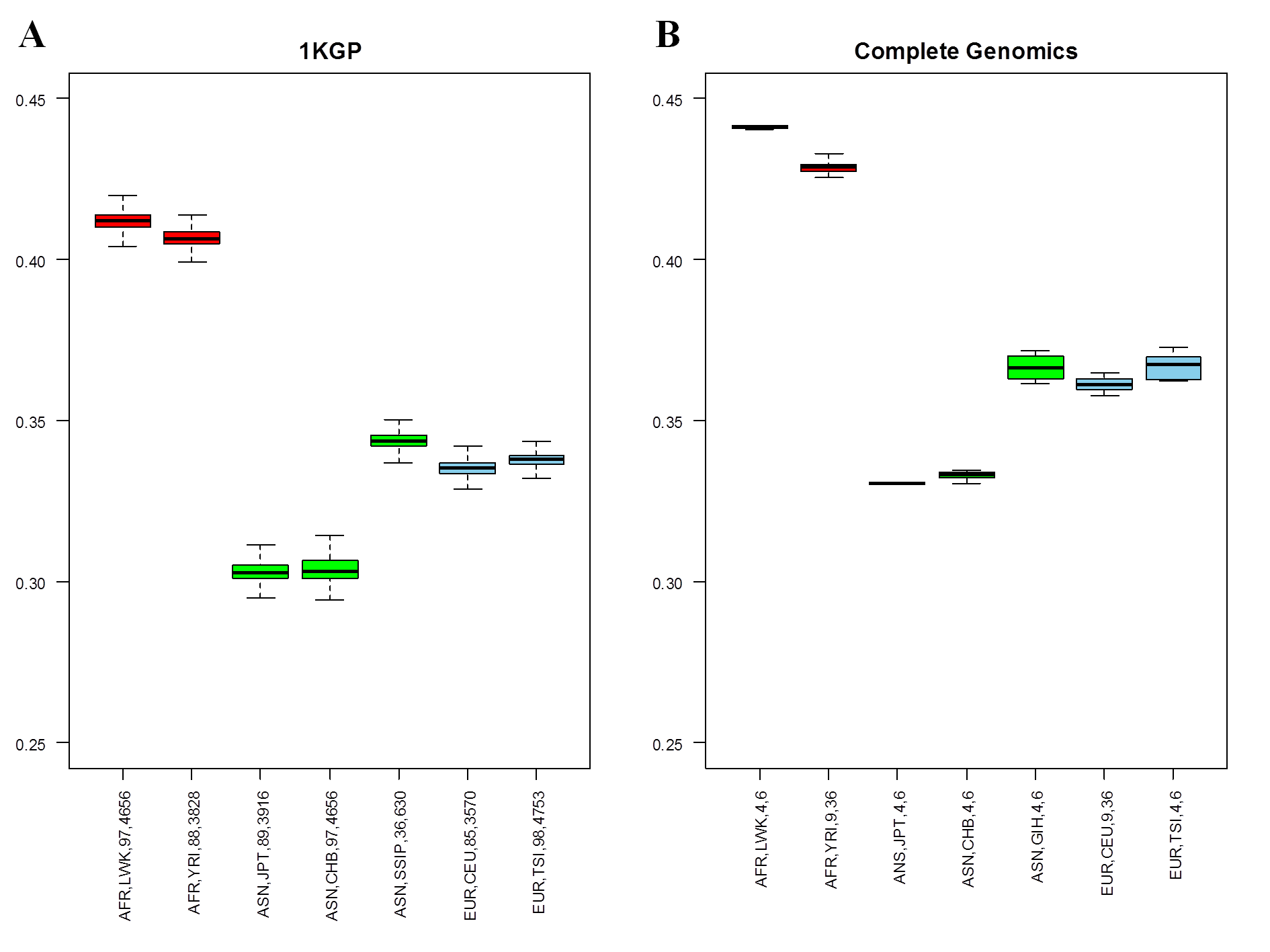

Supplement: Figure S9 — Intra population diversity for 7 populations in 1 KGP and Complete Genomics. Intra population diversity base on distance measure D for 7 populations for (A) 1 KGP (average coverage of 5X) and (B) Complete Genomics (deep coverage of 51-89X). Label at axis X show information of continent, population, sample size and total number of pair. Identical trend was observed regardless of low or deep sequencing coverage. African populations have the highest intra-population diversity score while Asian populations have the lowest, GIH (Northern Indians) and SSIP are slightly above Europeans. (TIF) [file pgen.1004377.s009.tif]

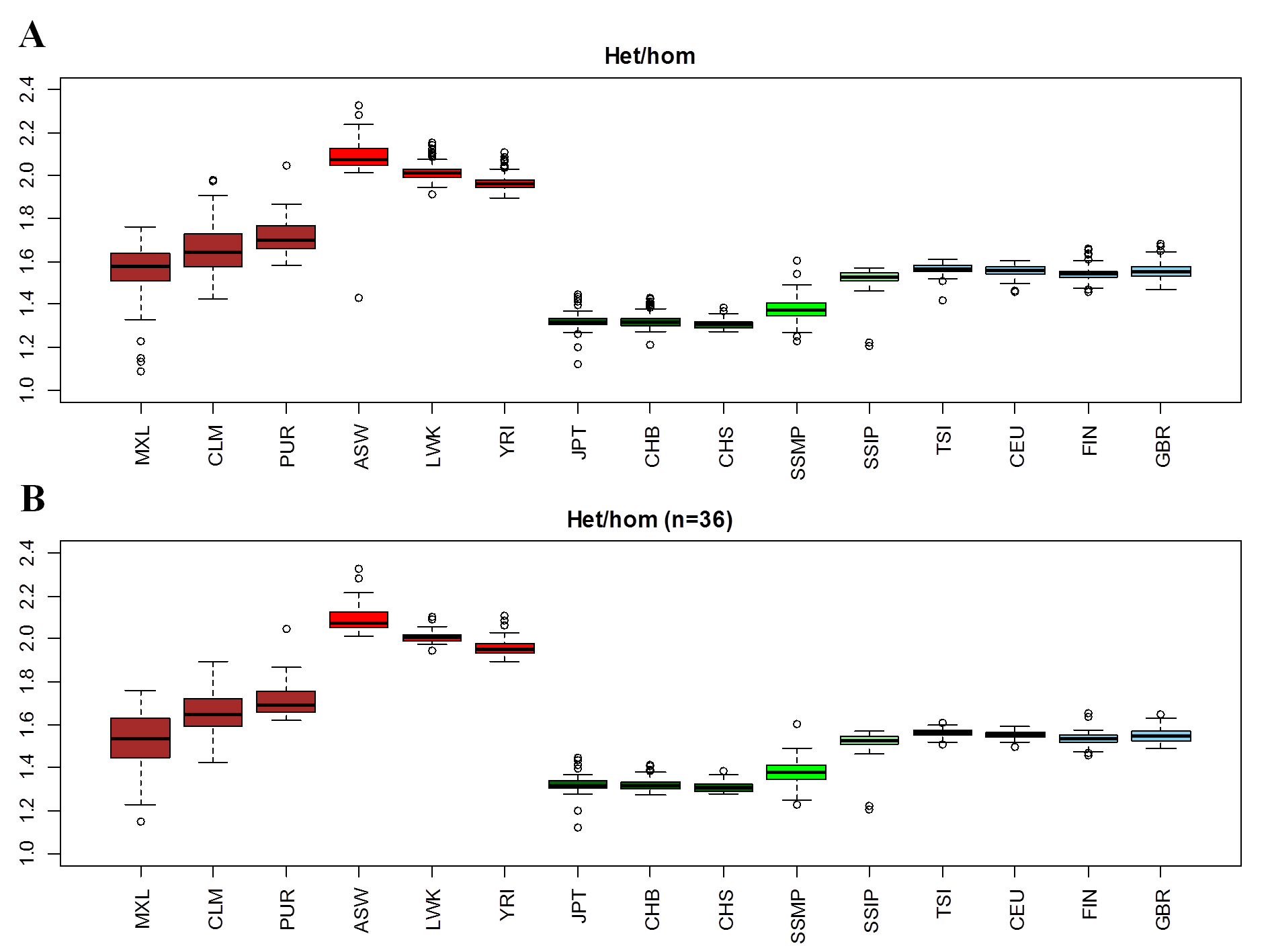

Supplement: Figure S10 — Heterozygous to homozygous ratio. (A) Boxplot of original sample sizes heterozygous to homozygous ratio in each population. (B) We randomly selected 36 samples from each population (SSIP, SSMP and 1 KGP) to calculate single sample heterozygous to homozygous ratio. SSIP has the highest ratio than all other Asian populations, indicating SSIP is more diverse than East Asian populations (JPT, CHB, CHS) and Southeast Asian population (SSMP). (TIF) [file pgen.1004377.s010.tif]

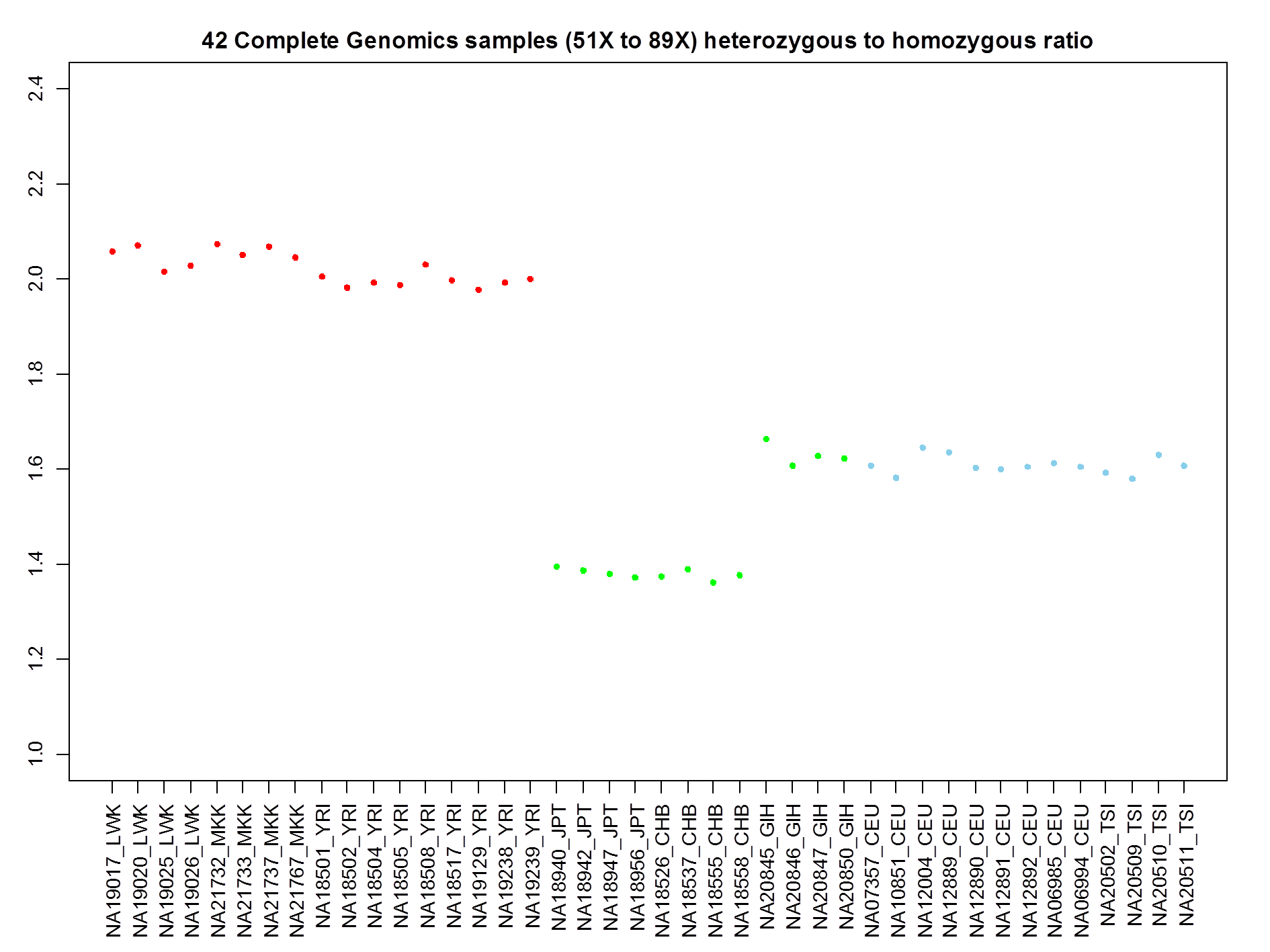

Supplement: Figure S11 — Heterozygous to homozygous ratio for Complete Genomics samples. Heterozygous to homozygous ratio for samples from Complete Genomics color coded by continent, red represents Africans, green for Asians while skyblue for Europeans. Het/Hom ratio obtained in deep sequencing samples (Complete Genomics) and shallow sequencing samples (1 KGP) are within the same ranges for all compatible populations. Axis X show information of sample id and population. (TIF) [file pgen.1004377.s011.tif]

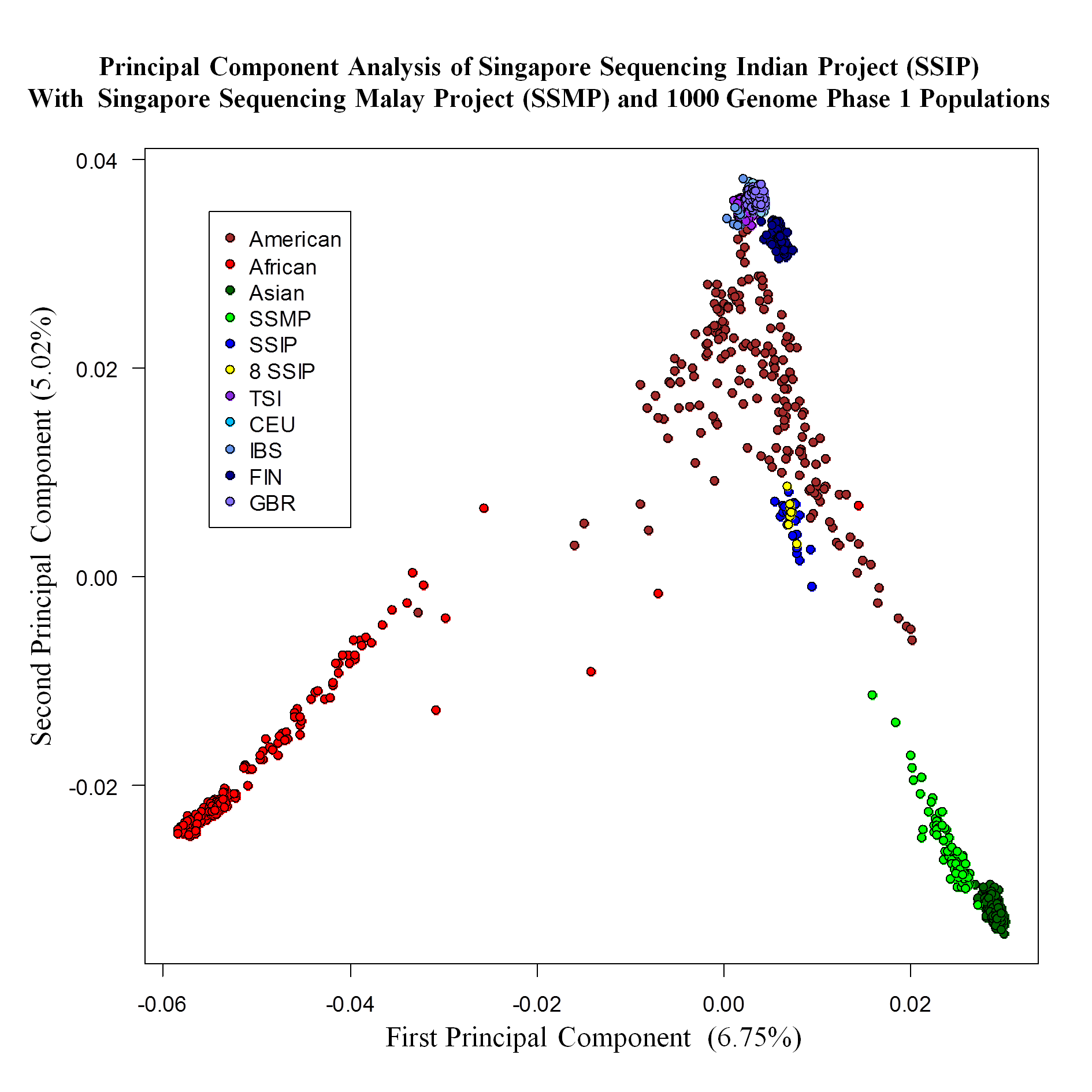

Supplement: Figure S12 — Principal component analysis of 16 world populations. PCA of 1,224 samples from SSIP, SSMP and 14 populations from Phase 1 of the 1 KGP, where the samples are grouped and color-coded by continents (legend). Blue circles are 26 SSIP samples and the remaining 8 SSIP individuals (yellow circles) are with European dominant mitochondria haplogroup assignment. (TIF) [file pgen.1004377.s012.tif]

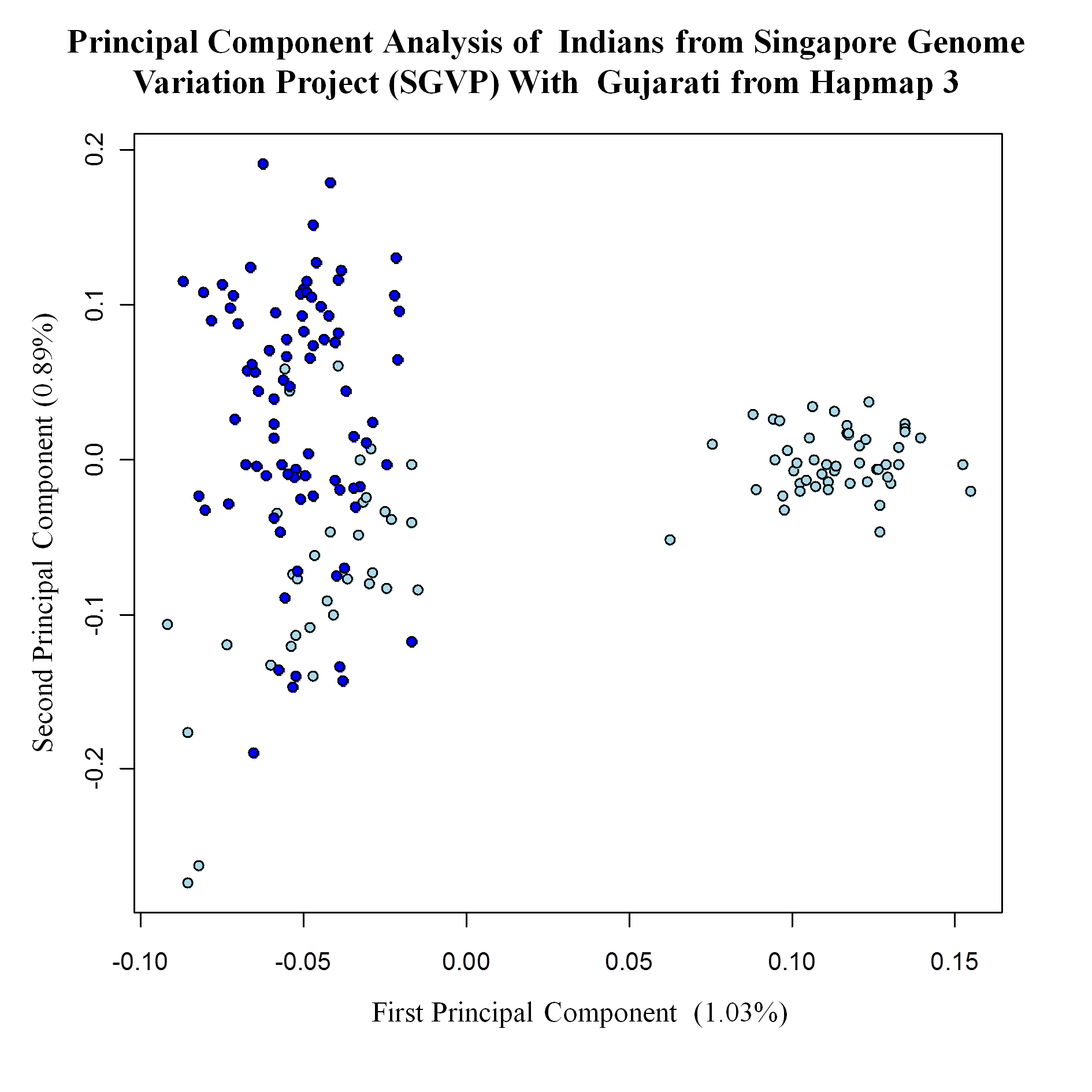

Supplement: Figure S13 — Principal component analysis of 83 Indians from Singapore Genome Variation Project and 85 Gujarathi from Hapmap 3. PCA on a set of 30,927 SNPs for 83 Singapore Indians (blue) from SGVP and 85 Gujarati Indians in Houston (skyblue) from Hapmap 3. (TIF) [file pgen.1004377.s013.tif]

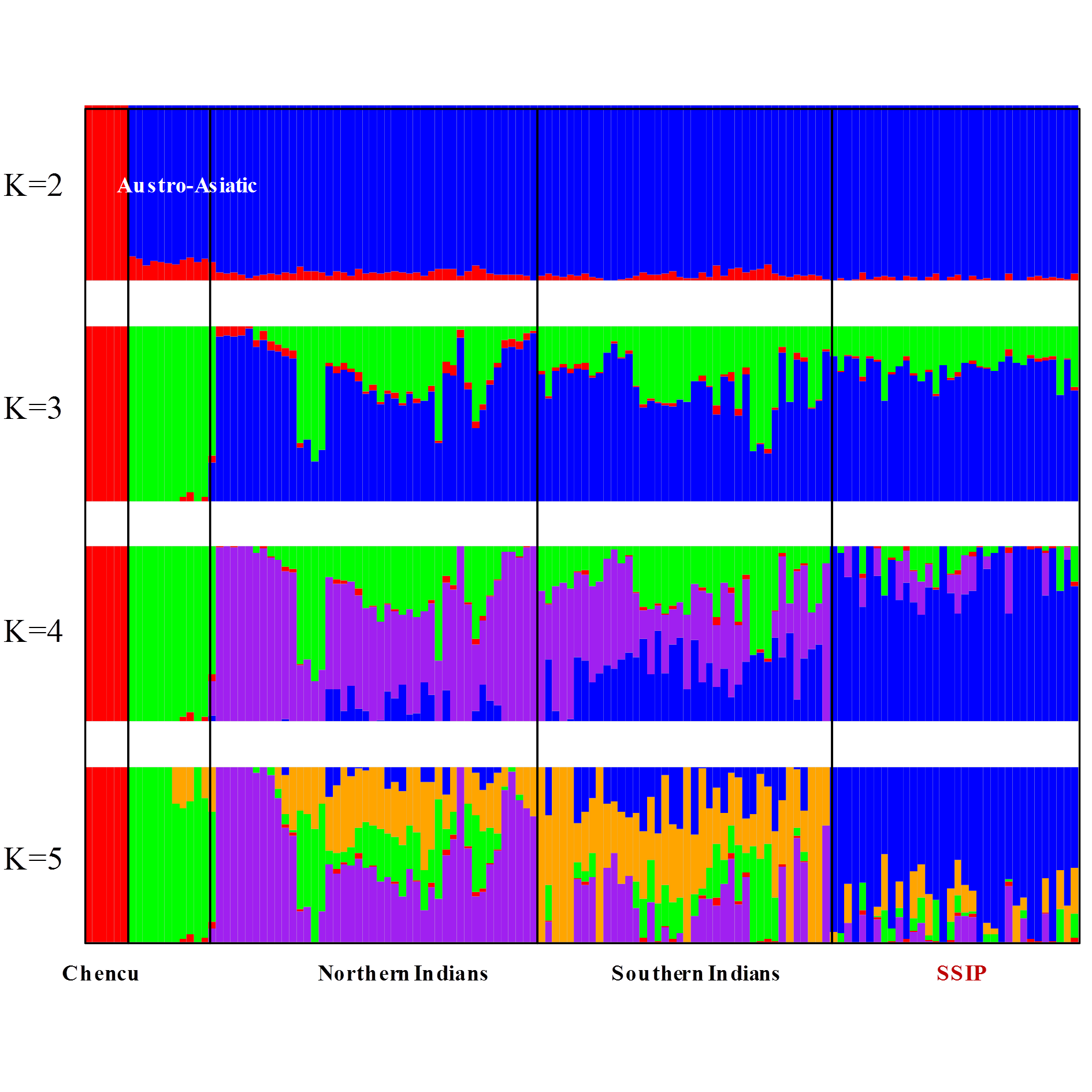

Supplement: Figure S14 — Admixture analysis on 104 samples from 20 Indian subgroups [11] and 36 SSIP individuals. An analysis of admixture on the 20 Indian subgroups and SSIP with ADMIXTURE program, K is the number of distinct populations that varied between 2 and 5. Black windows highlights the position of the Chencu, Austro-Asiatic groups (Kharia and Santhal), Northern Indians and Southern Indians (see Table S2 for the categorization of the 20 Indian subgroups into Southern or Northern Indians). (TIF) [file pgen.1004377.s014.tif]

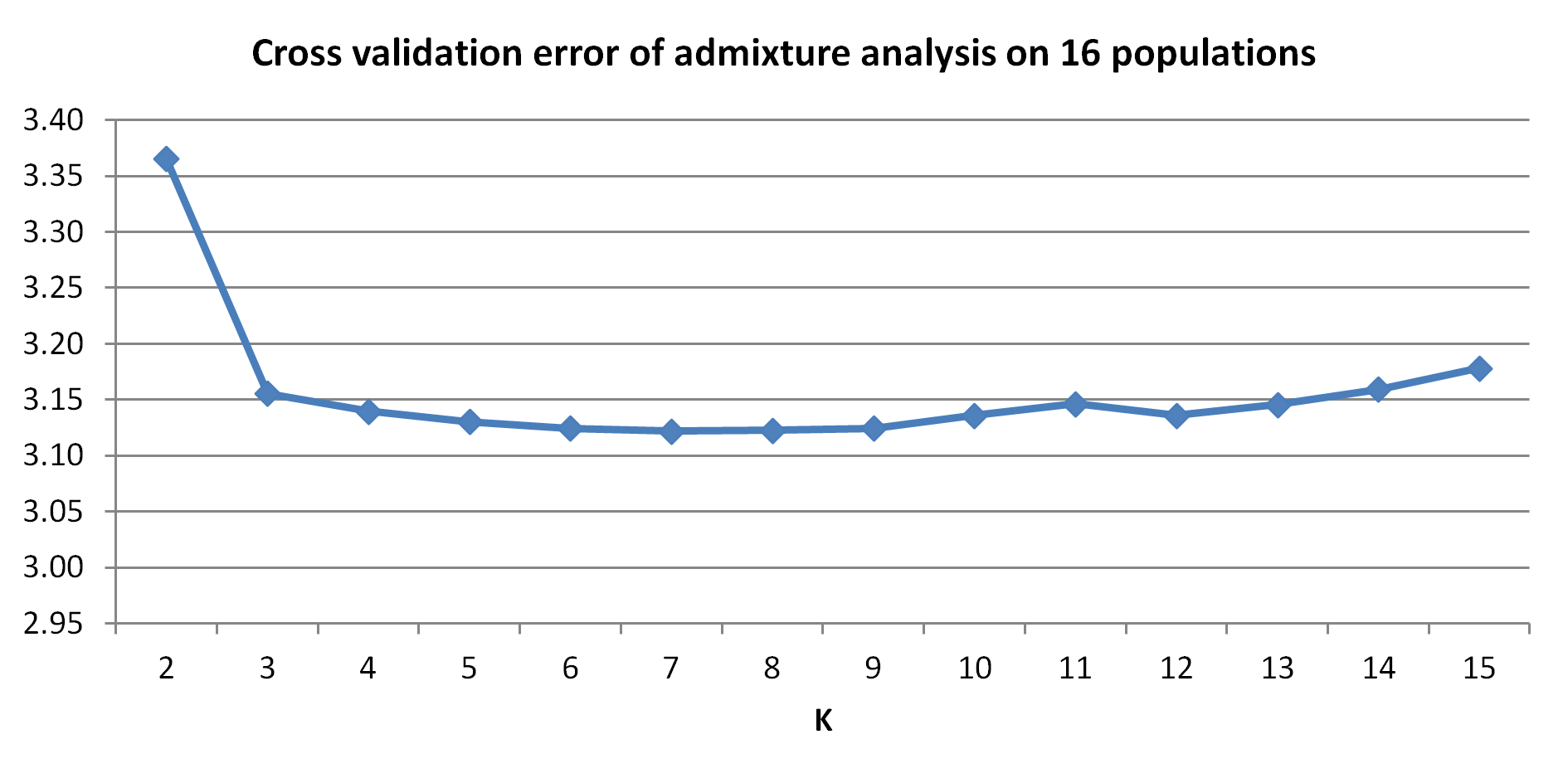

Supplement: Figure S15 — Cross validation error for admixture analysis with K from 2 to 15 performed on 16 populations. Cross-validation error in the admixture analysis at different values of ancestral groups (K), which we have allowed to range from 2 to 15. It was observed that while K = 7 yielded the lowest cross-validation error, K = 6 yielded a difference that was less than 0.01 and thus K = 6 was chosen for reporting in the main text on the basis of parsimony. (TIF) [file pgen.1004377.s015.tif]

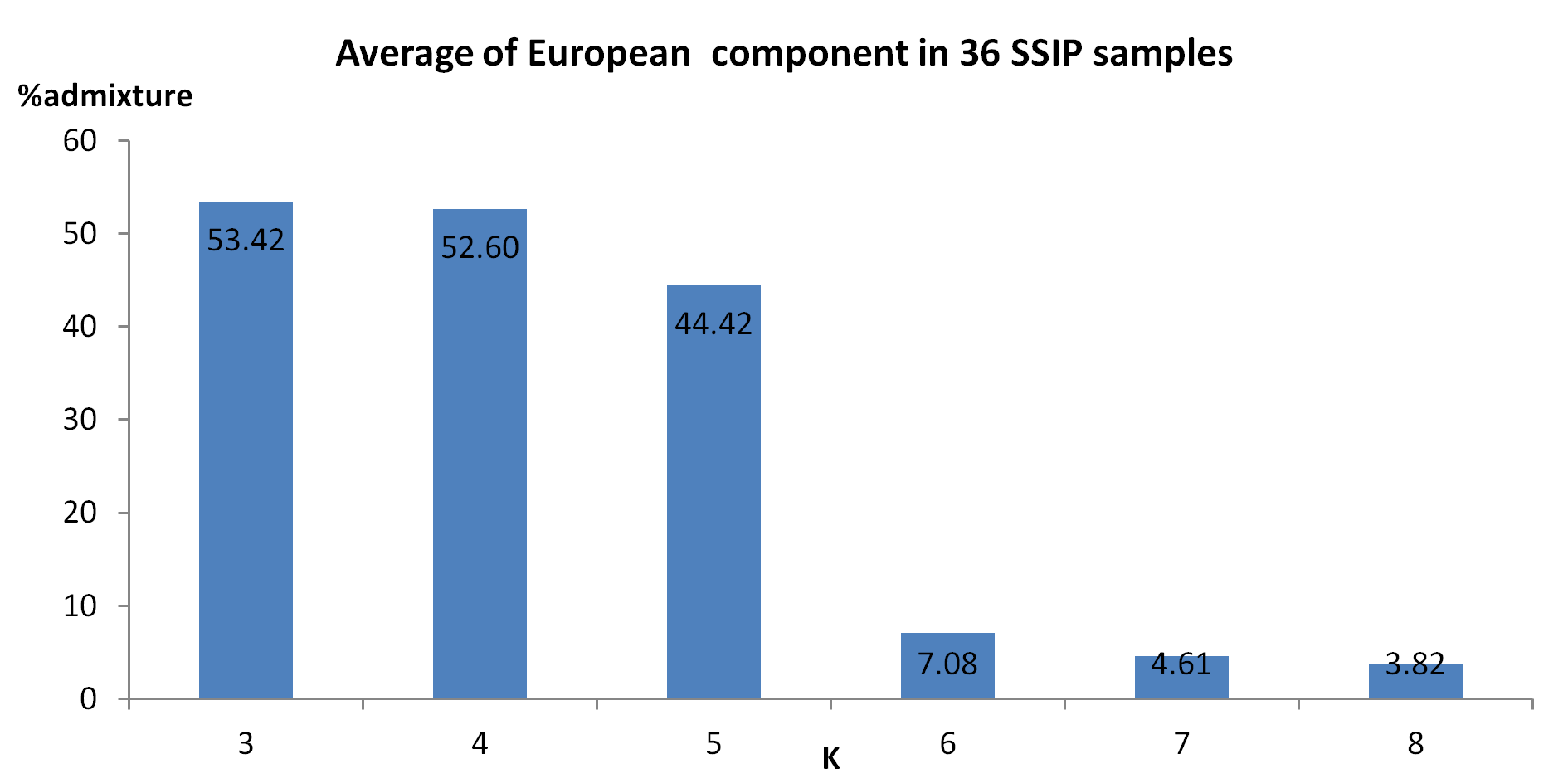

Supplement: Figure S16 — European component in 36 SSIP individuals from admixture analysis. Bars represent average of European component in 36 SSIP samples from the admixture analysis on 6,519,079 SNPs of 16 populations in which 14 populations from 1 KGP (Table S1), SSMP and SSIP. K indicates the number of ancestral populations. At K = 3, the three ancestral populations are African, European and Asian (Figure 3B) where an average of 53.42% European component was found in 36 SSIP individuals. When K increased, more ancestral populations contributed to the entire admixture panel that likely to dilute the European component possessed by SSIP individuals. (TIF) [file pgen.1004377.s016.tif]
