# Supplementary material for: Insights into the Genetic Structure and Diversity of 38 South Asian Indians from Deep Whole-Genome Sequencing
Source: PLoS Genet. 2014 May 15;10(5):e1004377. doi: 10.1371/journal.pgen.1004377 (PMC4022468; doi:10.1371/journal.pgen.1004377)
Supplement: Table S1 — Description of populations used for comparison with SSIP. (DOC) [file pgen.1004377.s017.doc]

**Table S1. Description of populations used for comparison with SSIP**

| **Project** | **Description** | **Number of sample** |
| --- | --- | --- |
| 1KGP |  |  |
| MXL | HapMap Mexican individuals from LA California | 66 |
| CLM | Colombian in Medellin, Colombia | 60 |
| PUR | Puerto Rican in Puerto Rico | 55 |
| ASW | HapMap African ancestry individuals from SW US | 61 |
| LWK | Luhya individuals | 97 |
| YRI | Yoruba individuals | 88 |
| JPT | Japanese individuals | 89 |
| CHB | Han Chinese in Beijing | 97 |
| CHS | Han Chinese South | 100 |
| TSI | Toscan individuals | 98 |
| CEU | CEPH individuals | 85 |
| IBS | Iberian populations in Spain | 14 |
| FIN | HapMap Finnish individuals from Finland | 93 |
| GBR | British individuals from England and Scotland | 89 |
| Complete Genomics |  |  |
| CG_LWK | Luhya in Webuye, Kenya | 4 |
| CG_YRI | Yoruba in Ibadan, Nigeria | 4 |
| CG_MKK | Maasai in Kinyawa, Kenya | 9 |
| CG_JPT | Japanese in Tokyo, Japan | 4 |
| CG_CHB | Han Chinese in Beijing, China | 4 |
| CG_GIH | Gujarati Indian in Houston, Texas, USA | 4 |
| CG_CEU | Utah residents with Northern and Western | 9 |
| CG_TSI | Toscans in Italy | 4 |
| SSMP |  |  |
| SSMP | Malays in Singapore | 96 |
