# Supplementary material for: Insights into the Genetic Structure and Diversity of 38 South Asian Indians from Deep Whole-Genome Sequencing
Source: PLoS Genet. 2014 May 15;10(5):e1004377. doi: 10.1371/journal.pgen.1004377 (PMC4022468; doi:10.1371/journal.pgen.1004377)
Supplement: Table S2 — Description of 25 Indian groups extracted from Reich et al. 2009. (DOC) [file pgen.1004377.s018.doc]

**Table S2. Description of 25 Indian groups extracted from Reich et al. 2009**

| **Group** | **Language Family** | **Latitude/longitude** | **North/South** |
| --- | --- | --- | --- |
| Kashmiri_Pandit | Indo-European | 34^○^22'N/75^○^50'E | North |
| Vaish | Indo-European | 25^○^46'N/82^○^44'E | North |
| Srivastava | Indo-European | 25^○^10'N/82^○^37'E | North |
| Sahariya | Indo-European | 25^○^28'N/81^○^54'E | North |
| Lodi | Indo-European | 26^○^45'N/83^○^24'E | North |
| Satnami | Indo-European | 20^○^29'N/85^○^58'E | North |
| Bhil | Indo-European | 23^○^02'N/72^○^40'E | North |
| Tharu | Indo-European | 29^○^23'N/27^○^30'E | North |
| Meghawal | Indo-European | 26^○^18'N/73^○^04'E | North |
| Kharia | Austro-Asiatic | 23^○^08'N/73^○^07'E | North |
| Nyshi | Tibeto-Burman | 26^○^55'N/92^○^40'E | North |
| Aonaga | Tibeto-Burman | 25^○^40'N/94^○^08'E | North |
| Santhal | Austro-Asiatic | 24^○^30'N/87^○^30'E | North |
| Vysya | Dravidian | 14^○^14'N/77^○^39'E | South |
| Naidu | Dravidian | 13^○^13'N/79^○^06'E | South |
| Velama | Dravidian | 16^○^31'N/75^○^51'E | South |
| Madiga | Dravidian | 17^○^58'N/79^○^35'E | South |
| Mala | Dravidian | 17^○^22'N/78^○^29'E | South |
| Kamsali | Dravidian | 15^○^49'N/78^○^02'E | South |
| Chenchu | Dravidian | 17^○^22'N/78^○^28'E | South |
| Kurumba | Dravidian | 10^○^54'N/76^○^27'E | South |
| Hallaki | Dravidian | 13^○^55'N/74^○^09'E | South |
| Siddi | Dravidian | 15^○^17'N/75^○^05'E | South |
| Onge | Jarawa-Onge | 10^○^30'N/92^○^30'E | South |
| Great_Andamanese | Andamanese | 12^○^12'N/93^○^00'E | South |
