# Supplementary material for: Insights into the Genetic Structure and Diversity of 38 South Asian Indians from Deep Whole-Genome Sequencing
Source: PLoS Genet. 2014 May 15;10(5):e1004377. doi: 10.1371/journal.pgen.1004377 (PMC4022468; doi:10.1371/journal.pgen.1004377)
Supplement: Table S3 — Transition to transversion ratio (Ts/Tv). (A) Ts/Tv for bi-allelic SNPs, (B) Ts/Tv after the removal of CpG exonic transition SNPs. (DOC) [file pgen.1004377.s019.doc]

**Table S3A. Transition to transversion ratio (Ts/Tv) for bi-allelic SNPs**

|  | **Whole genome** | | | **Exome** | | |
| --- | --- | --- | --- | --- | --- | --- |
|  | Known | Novel | Total | Known | Novel | Total |
| Transition (Ts) | 5,598,290 | 1,410,579 | 7,008,869 | 43,840 | 12,454 | 56,294 |
| Transversion (Tv) | 2,596,828 | 680,813 | 3,277,641 | 12,808 | 4,551 | 17,359 |
| Ts/Tv | 2.16 | 2.07 | 2.14 | 3.42 | 2.74 | 3.24 |

**Table S3B. Ts/Tv ratio after the removal of CpG exonic transition SNPs**

| **SNP type** | **Nucleotide Changes** | **#SNPs** | **Subtotal** |
| --- | --- | --- | --- |
| Ts | C->T | 8,074 |  |
|  | T->C | 9,190 |  |
|  | A->G | 9,115 |  |
|  | G->A | 8,154 | 34,533 |
| Tv | A->C | 1,669 |  |
|  | A->T | 1,101 |  |
|  | C->A | 2,600 |  |
|  | G->T | 2,532 |  |
|  | T->A | 1,076 |  |
|  | T->G | 1,683 |  |
|  | C->G | 3,115 |  |
|  | G->C | 3,223 | 16,999 |
| Ts/Tv | | | 2.03 |
