# Supplementary material for: Insights into the Genetic Structure and Diversity of 38 South Asian Indians from Deep Whole-Genome Sequencing
Source: PLoS Genet. 2014 May 15;10(5):e1004377. doi: 10.1371/journal.pgen.1004377 (PMC4022468; doi:10.1371/journal.pgen.1004377)
Supplement: Table S4 — Summary of single sample Loss-of-function (LOF) variants. (DOC) [file pgen.1004377.s020.doc]

**Table S4. Summary of single sample Loss-of-function (LOF) variants**

|  | **NOVEL** | | | **KNOWN** | | | **ALL** | | |
| --- | --- | --- | --- | --- | --- | --- | --- | --- | --- |
|  | Low | Common | Total | Low | Common | Total | Low | Common | Total |
| Mean | 6.86 | 23.08 | 29.94 | 6.97 | 313.83 | 320.81 | 13.83 | 336.92 | 350.75 |
| Standard deviation | 2.37 | 3.48 | 3.88 | 3.09 | 8.39 | 8.31 | 4.05 | 8.44 | 8.56 |
| Maximum | 11 | 31 | 38 | 15 | 328 | 335 | 25 | 354 | 368 |
| Minimum | 1 | 16 | 22 | 1 | 295 | 304 | 6 | 318 | 333 |
| Quartile 25% | 5 | 20 | 27 | 5 | 306 | 314 | 11 | 330 | 345 |
| Median | 6 | 23 | 29 | 7 | 314 | 320 | 13 | 335 | 350 |
| Quartile 75% | 8 | 25 | 32 | 9 | 320 | 326 | 16 | 342 | 357 |
| Total | 228 | 407 | 635 | 161 | 633 | 794 | 389 | 1,040 | 1,429 |
