# Supplementary material for: Insights into the Genetic Structure and Diversity of 38 South Asian Indians from Deep Whole-Genome Sequencing
Source: PLoS Genet. 2014 May 15;10(5):e1004377. doi: 10.1371/journal.pgen.1004377 (PMC4022468; doi:10.1371/journal.pgen.1004377)
Supplement: Table S5 — List of pathways affected by Loss-of-function (LOF) variants. (DOC) [file pgen.1004377.s021.doc]

**Table S5. List of pathways affected by LOF variants**

| **KEGG pathway** | **# genes** | **P-value** | **Benjamini** |
| --- | --- | --- | --- |
| Olfactory transduction | 28 | 2.5E-5 | 2.8E-3 |
| ABC transporters | 6 | 1.1E-2 | 4.5E-1 |
| Histidine metabolism | 4 | 5.8E-2 | 8.9E-1 |
