# Supplementary material for: Insights into the Genetic Structure and Diversity of 38 South Asian Indians from Deep Whole-Genome Sequencing
Source: PLoS Genet. 2014 May 15;10(5):e1004377. doi: 10.1371/journal.pgen.1004377 (PMC4022468; doi:10.1371/journal.pgen.1004377)
Supplement: Table S6 — List of Loss-of-function (LOF) variants found in COSMIC database. (DOC) [file pgen.1004377.s022.doc]

**Table S6. List of LOF variants found in COSMIC database**

| **SNP** | **Site** | **Gene** | **Allele Frequency in SSIP*** |
| --- | --- | --- | --- |
| rs11303415  rs144690814  rs201917032  rs34061715  rs61397759 | Ovary | RHBG  OR8I2  AQP12B  HRNR  LOC148213 | 0.64 (n=22)  0.50 (n=2)  0.50 (n=9)  1.00 (n=36)  0.55 (n=11) |
| rs201644946  rs142406301  rs148800656  rs35342965 | Gastrointestinal | PROL1  RECQL5  ZAN  MMP12 | 0.06 (n=36)  0.50 (n=26)  0.64 (n=25)  1.00 (n=36) |
| rs75266995 | Liver | OR7G3 | 0.55 (n=11) |
| rs71818662 | Pancreas | IL32 | 0.12 (n=4) |

*n is the number of sample in SSIP having that particular SNP
