# Supplementary material for: Insights into the Genetic Structure and Diversity of 38 South Asian Indians from Deep Whole-Genome Sequencing
Source: PLoS Genet. 2014 May 15;10(5):e1004377. doi: 10.1371/journal.pgen.1004377 (PMC4022468; doi:10.1371/journal.pgen.1004377)
Supplement: Table S7 — List of Loss-of-function (LOF) variants related to GWAS studies. (DOC) [file pgen.1004377.s023.doc]

**Table S7. List of LOF variants related to GWAS studies**

| **SNP** | **Catalogue Id*** | **Genes** | **Trait** | **Allele Frequency in SSIP^#^** |
| --- | --- | --- | --- | --- |
| rs1861050 | rs1861050 | KIAA1345 | Conduct disorder (case status) | 0.10 (n=36) |
| rs328 | rs328 | LPL | Triglycerides | 0.06 (n=36) |
| rs328 | rs328 | LPL | HDL cholesterol | 0.06 (n=36) |
| rs61735710 | rs1048886 | C6orf57 | Type 2 diabetes | 0.01 (n=36) |

*SNP id as reported by the cited source, rs61735710 is one nucleotide upstream to rs1048886.

^#^ n is number of sample in SSIP having that particular SNP
