# Supplementary material for: Insights into the Genetic Structure and Diversity of 38 South Asian Indians from Deep Whole-Genome Sequencing
Source: PLoS Genet. 2014 May 15;10(5):e1004377. doi: 10.1371/journal.pgen.1004377 (PMC4022468; doi:10.1371/journal.pgen.1004377)
Supplement: Table S8 — The proportions for 6 ancestral populations extracted from the output of ADMIXTURE program. (DOC) [file pgen.1004377.s024.doc]

**Table S8. The proportions for 6 ancestral populations extracted from the output of ADMIXTURE program**

| **Sample** | **Gender*** | **chrM Haplogroup** | **chrY Haplogroup** | **African** | **North East Asian** | **South East Asian** | **European** | **American** | **South Asian** |
| --- | --- | --- | --- | --- | --- | --- | --- | --- | --- |
| SSI004 | M | F1c1a | J2b2 | 0.0037 | 0.0352 | 0.0303 | 0.0730 | 0.0143 | 0.8436 |
| SSI024 | F | HV12b |  | 0.0000 | 0.0177 | 0.0380 | 0.1021 | 0.0137 | 0.8284 |
| SSI030 | F | HV12b |  | 0.0000 | 0.0000 | 0.0529 | 0.1649 | 0.0111 | 0.7710 |
| SSI013 | F | HV14 |  | 0.0000 | 0.0442 | 0.0000 | 0.0772 | 0.0002 | 0.8784 |
| SSI006 | M | M | L1 | 0.0000 | 0.0151 | 0.0360 | 0.0698 | 0.0042 | 0.8749 |
| SSI012 | M | M2a'b | L1 | 0.0000 | 0.0341 | 0.0153 | 0.0686 | 0.0100 | 0.8720 |
| SSI003 | F | M30d |  | 0.0000 | 0.0000 | 0.0289 | 0.1096 | 0.0000 | 0.8615 |
| SSI005 | F | M33a+146 |  | 0.0000 | 0.0000 | 0.0394 | 0.1269 | 0.0100 | 0.8237 |
| SSI020 | F | M34 |  | 0.0000 | 0.0000 | 0.0000 | 0.0000 | 0.0000 | 1.0000 |
| SSI027 | F | M34 |  | 0.0000 | 0.0000 | 0.0000 | 0.0000 | 0.0000 | 1.0000 |
| SSI026 | F | M35a1 |  | 0.0000 | 0.0000 | 0.0271 | 0.0630 | 0.0088 | 0.9011 |
| SSI028 | F | M35a1 |  | 0.0000 | 0.0143 | 0.0541 | 0.0406 | 0.0090 | 0.8820 |
| SSI022 | F | M36 |  | 0.0000 | 0.0000 | 0.0647 | 0.0375 | 0.0048 | 0.8931 |
| SSI010 | F | M36d1 |  | 0.0048 | 0.0037 | 0.0512 | 0.1057 | 0.0000 | 0.8346 |
| SSI031 | M | M3a1 | J2b2 | 0.0000 | 0.0000 | 0.0146 | 0.0564 | 0.0000 | 0.9289 |
| SSI014 | M | M3a1 | L1 | 0.0000 | 0.0311 | 0.0429 | 0.0934 | 0.0018 | 0.8308 |
| SSI015 | F | M3a2 |  | 0.0000 | 0.0000 | 0.0380 | 0.0568 | 0.0277 | 0.8776 |
| SSI018 | F | M40 |  | 0.0000 | 0.0010 | 0.0284 | 0.0485 | 0.0116 | 0.9104 |
| SSI037 | F | M40a |  | 0.0000 | 0.0064 | 0.0529 | 0.1092 | 0.0000 | 0.8315 |
| SSI009 | M | M53 | H1 | 0.0000 | 0.0192 | 0.0345 | 0.1048 | 0.0000 | 0.8414 |
| SSI038 | F | M5a2a |  | 0.0000 | 0.0021 | 0.0712 | 0.0000 | 0.0000 | 0.9267 |
| SSI036 | M | M5c1 | L1 | 0.0000 | 0.0000 | 0.0351 | 0.0669 | 0.0123 | 0.8857 |
| SSI011 | F | M66 |  | 0.0000 | 0.0314 | 0.0489 | 0.0000 | 0.0000 | 0.9197 |
| SSI039 | F | M66 |  | 0.0000 | 0.0394 | 0.0000 | 0.0722 | 0.0000 | 0.8884 |
| SSI021 | F | M6a1 |  | 0.0000 | 0.0000 | 0.0364 | 0.0647 | 0.0180 | 0.8809 |
| SSI040 | F | M6a1 |  | 0.0000 | 0.0101 | 0.0657 | 0.0107 | 0.0077 | 0.9058 |
| SSI019 | F | M7c3c |  | 0.0092 | 0.0000 | 0.2128 | 0.0491 | 0.0000 | 0.7290 |
| SSI008 | F | N8 |  | 0.0000 | 0.0375 | 0.1285 | 0.0953 | 0.0097 | 0.7291 |
| SSI029 | M | R6 | H1 | 0.0000 | 0.0404 | 0.0025 | 0.1029 | 0.0000 | 0.8541 |
| SSI035 | F | R6a |  | 0.0000 | 0.0270 | 0.0026 | 0.0747 | 0.0039 | 0.8918 |
| SSI034 | M | R8a1a1 | H1a1 | 0.0000 | 0.0093 | 0.0626 | 0.1199 | 0.0000 | 0.8082 |
| SSI017 | F | T2a1a |  | 0.0000 | 0.0000 | 0.0974 | 0.0554 | 0.0099 | 0.8373 |
| SSI032 | M | U1a3 | R1a1 | 0.0000 | 0.0000 | 0.0714 | 0.0907 | 0.0072 | 0.8306 |
| SSI033 | F | U1a3 |  | 0.0000 | 0.0274 | 0.0062 | 0.0899 | 0.0000 | 0.8764 |
| SSI025 | F | U7 |  | 0.0000 | 0.0202 | 0.0256 | 0.0982 | 0.0086 | 0.8473 |
| SSI023 | M | W3a1 | BCDEF | 0.0000 | 0.0046 | 0.0434 | 0.0512 | 0.0220 | 0.8788 |

*F: female; M: male
