# Supplementary material for: Insights into the Genetic Structure and Diversity of 38 South Asian Indians from Deep Whole-Genome Sequencing
Source: PLoS Genet. 2014 May 15;10(5):e1004377. doi: 10.1371/journal.pgen.1004377 (PMC4022468; doi:10.1371/journal.pgen.1004377)
Supplement: Table S9 — D statistic analysis with ancient genome for 5 randomly selected pairwise samples, anchored with different SSIP sample (G1). (A) Neanderthal as ancient hominid, (B) Denisovan as ancient hominid. (DOC) [file pgen.1004377.s025.doc]

**Table S9A. D statistic analysis with Neanderthal genome for 5 randomly selected pairwise samples, anchored with different SSIP sample (G1)**

| **Population** | **1KGP (2-6X)** |  |  | **Complete Genomics** | **(51-89X)** |
| --- | --- | --- | --- | --- | --- |
| **G2** | **Mean %Dstatisitc** | **Standard deviation** |  | **Mean %Dstatisitc** | **Standard deviation** |
|  |  | **Africans** |  |  |  |
| LWK | 2.5654 | 0.29 |  | 2.9140 | 0.65 |
| YRI | 2.9993 | 0.31 |  | 2.9243 | 0.50 |
|  |  | **Asians** |  |  |  |
| JPT | -0.8431 | 0.48 |  | -0.8232 | 0.71 |
| CHB | -1.0029 | 0.69 |  | -0.7378 | 0.38 |
|  |  | **Europeans** |  |  |  |
| CEU | -0.2317 | 0.08 |  | -0.2356 | 0.20 |

**Table S9B. D statistic analysis with Denisovan genome for 5 randomly selected pairwise samples anchored with different SSIP sample (G1)**

| **Population** | **1KGP (2-6X)** |  |  | **Complete Genomics** | **(51-89X)** |
| --- | --- | --- | --- | --- | --- |
| **G2** | **Mean %Dstatisitc** | **Standard deviation** |  | **Mean %Dstatisitc** | **Standard deviation** |
|  |  | **Africans** |  |  |  |
| LWK | -1.6356 | 0.47 |  | -0.7275 | 0.27 |
| YRI | -0.9381 | 0.12 |  | -0.9315 | 0.67 |
|  |  | **Asians** |  |  |  |
| JPT | 0.7085 | 0.67 |  | 0.7052 | 0.53 |
| CHB | -0.9975 | 0.81 |  | -0.8747 | 0.40 |
|  |  | **Europeans** |  |  |  |
| CEU | 0.5491 | 0.47 |  | 0.5932 | 0.47 |
