# Supplementary material for: Insights into the Genetic Structure and Diversity of 38 South Asian Indians from Deep Whole-Genome Sequencing
Source: PLoS Genet. 2014 May 15;10(5):e1004377. doi: 10.1371/journal.pgen.1004377 (PMC4022468; doi:10.1371/journal.pgen.1004377)
Supplement: Text S1 — Supplementary methods. (DOC) [file pgen.1004377.s026.doc]

**Text S1**

**INSIGHTS INTO THE GENETIC STRUCTURE AND DIVERSITY OF 38 SOUTH ASIAN INDIANS FROM DEEP WHOLE GENOME SEQUENCING**

Lai-Ping Wong, Jason Kuan-Han Lai, Woei-Yuh Saw, Rick Twee-Hee Ong, Anthony Youzhi Cheng, Nisha Esakimuthu Pillai, Xuanyao Liu, Wenting Xu, Peng Chen, Jia-Nee Foo, Linda Wei-Lin Tan, Seok-Hwee Koo, Richie Soong, Markus Rene Wenk, Wei-Yen Lim, Chiea-Chuen Khor, Peter Little, Kee-Seng Chia, Yik-Ying Teo

**CONTENTS**

**1 Methods** 2

1.1 Accessible genome measurement 2

1.2 SNPs discovery 2

1.3 Genotype concordance between Sequencing and Omni 2.5 M genotyping 4

1.4 Transition and transversion SNPs 4

1.5 Functional annotation of SNPs 4

1.6 Indels discovery and functional annotation 4

1.7 Structural variation (deletion) discovery 5

1.7.1 BreakDancer deletion discovery 5

1.7.2 VariationHunter deletion discovery 6

1.7.3 Pindel deletion discovery 6

1.7.4 Delly deletion discovery 7

1.8 Haplotype phasing of the SSIP genotype data 7

1.9 Mitochondria haplogroup Assignment 7

1.10 Chromosome Y haplogroup Assignment 7

**2 References** 9

**1 Methods**

**1.1 Accessible genome measurement**

We measured accessible genome by taking proportion of pass quality control properly paired reads that were successfully mapped to the human reference genome NCBI build 37. Sequence reads were filtered with SAMTOOLS 0.1.18 [27] to remove PCR duplicates, and subsequently the BEDTools version 1.18.0 was used to calculate the accessible genome for each sample with the following command line:

samtools view -f 0x2 -b  [sample].bam  | samtools view -b -F 0x200 - | samtools view -b -F 0x400 - |genomeCoverageBed -d -ibam stdin | gzip -cv

In-house scripts were then used for calculating statistical summaries including quartile information on coverage, total coverage of mapped bases and total number of bases with at least 1 read coverage for mapped bases in each sample from the output of genomeCoverageBed. These metrics were summarized to calculate the average percentage of accessible genome across all SSIP samples.

**1.2 SNPs discovery**

We used two approaches to perform the SNP discovery process: (i) single-sample SNP calling using CASAVA; (ii) multi-sample SNP calling using The Genome Analysis Toolkit (GATK version 2.1.8) [28,29]

Variant calling in CASAVA uses callSmallVariants module that involve two stages, (i) Allele calling according to the sequence base calls, alignment scores (at least 6) and quality scores. Bases calles and their associated quality values were sent to Bayesian allele caller which output one or two allele calls and scores for each position in the genome. (ii) SNPs calling base on the basis of the allele calls and the read depth.

Allele calling considers the paired-end reads that satisfy all of the following criteria:

- Properly aligned and mapped to the reference genome;
- Pass the quality control filtering;
- Alignment score of at least 6;
- Read-pairs must map with the expected size and orientation.

CASAVA excludes reads that fail primary analysis quality checks which can be any of the following reads:

- PCR duplicates;
- optical duplicates;
- reads with a paired-end mapping quality score of less than 90.

Q(snp) and Q(max_gt) are two quality scores in the probabilistic model adopted by CASAVA in calculating the likelihood of the possible genotypes for every site in the genome. Q(snp) expresses the probability that the site will contain a variant allele while Q(max_gt) shows the probability of the most likely genotype at this site.

We retained candidate SNPs with Q(snp)>=20 and base depth smaller than 3 times the mean sequencing depth of the chromosome (to removes SNPs at regions close to centromeres with high copy numbers). Heterozygous SNPs on non-autosomal haploid chromosomes are also removed.

We used GATK’s UnifiedGenotyper for the multi-sample calling with the following workflow:

Step 1: Preprocessing

Customized script was used to replace bam file header and split the bam file by lane.

Step 2: Preparation of indel realigned bam file (for each sample)

% java -Xmx2g -jar $GATK -T RealignerTargetCreator -I $SAMPLE.bam -R $REF -o

$SAMPLE.intervals

% java -Xmx2g -jar $GATK -T IndelRealigner -I $SAMPLE.bam -R $REF -targetIntervals $SAMPLE.intervals -o SAMPLE.realign.bam

% SAMTOOLS index $SAMPLE.realign.bam

Step 3: Duplication Marking

% java -Xmx2g -jar $PICARD/MarkDuplicates.jar VALIDATION_STRINGENCY=SILENT

INPUT=SAMPLE.realign.bam OUTPUT=SAMPLE.realign.dedup.

bam METRICS_FILE=SAMPLE.metrics

% SAMTOOLS index SAMPLE.realign.dedup.bam

Step 4: Bases recalibration

% java -Xmx2g -jar $GATK -T BaseRecalibrator -I SAMPLE.realign.dedup.bam -R

REF -knownSites $DBSNP -o $SAMPLE.grp

% java -Xmx2g -jar $GATK -T PrintReads -I SAMPLE.realign.dedup.bam -R REF -

BQSR SAMPLE.grp -o SAMPLE.realign.dedup.recal.bam

% samtools index SAMPLE.realign.dedup.recal.bam

Step 5: Variant calling for SNPs

% java -Xmx4g -jar $GATK -nt 2 -T UnifiedGenotyper INPUT -R $REF -o indian.ug.raw.snp.vcf --genotype_likelihoods_model SNP

Step 6: SNPs recalibration

% java -Xmx4g -jar $GATK -T VariantRecalibrator -R REF \

-input NAME.ug.raw.snp.vcf \

-resource:hapmap,known=false,training=true,truth=true,prior=15.0

HAPMAP \

-resource:omni,known=false,training=true,truth=false,prior=11.0 OMNI \

-resource:dbsnp,known=true,training=false,truth=false,prior=6.0 DBSNP \

-an QD \

-an HaplotypeScore \

-an MQRankSum \

-an ReadPosRankSum \

-an FS \

-an MQ \

-an InbreedingCoeff \

-an DP \

-mode SNP \

-recalFile NAME.ug.snp.recal \

-tranchesFile NAME.ug.snp.tranches \

-rscriptFile NAME.ug.snp.plots.R

% java -Xmx2g -jar $GATK -T ApplyRecalibration -R REF \

-input NAME.ug.raw.snp.vcf \

--ts_filter_level 99.0 \

-tranchesFile NAME.ug.snp.tranches \

-recalFile NAME.ug.snp.recal \

-mode SNP \

-o $NAME.ug.filter.snp.vcf

To reduce the likelihood of false positives in the SNP calling, the final set of SNPs that are reported in the main text only included those that have been discovered by both CASAVA and GATK.

**1.3 Genotype concordance between Sequencing and Omni 2.5 M genotyping**

Each of the 38 SSIP samples has also been genotyped on the Illumina Human Omni 2.5 M genotyping array. Genotype data from this array were assessed for the following criteria:

- High rates of missingness (> 5%);
- Excess heterozygosity, which can be indicative of sample contamination;
- Excess identity-by-state, which suggests related or duplicated samples;
- Discordant ethnic membership from the reported ethnicity;
- Gender discrepancy.

We removed SNP if any one of the following conditions was met:

- Invalid chromosomal positions or strand;
- Duplicated chromosomal positions;
- High rates of missingness (call rate < 95%);
- Hardy-Weinberg equilibrium p-value < 10^-4^.

The SNPs on the Illumina Omni 2.5 M that passed the QC checks were used as gold standard to assess the accuracy of the genotype calls made by CASAVA and GATK on the sequence data.

**1.4 Transition and transversion SNPs**

A transition SNP refers to a variant that changes a purine nucleotide to another purine (A <-> G), or a pyrimidine nucleotide to another pyrimidine (C <-> T). A transversion SNP is a point mutation that changes a purine to a pyrimidine, or vice versa (A <-> C, A <-> T, C <-> G, G <-> T). The ratio of the number of transition SNPs to the number of transversion SNPs across whole genome bi-allelic SNPs is denoted as transition and transversion ratio (Ti/Tv).

**1.5 Functional annotation of SNPs**

Functional annotation of bi-allelic SNPs is performed using SNPEff [17]. Input file preparation and annotation results were summarized using customized computing scripts. The annotation results were binned into a list of categories and functional impacts as described in SNPEff website (http://snpeff.sourceforge.net/SnpEff_manual.html#eff)

**1.6 Indels discovery and functional annotation**

Similar with SNPs discovery, two approaches were used for indels calling. Workflow for indels calling is similar to SNPs calling (step 1 to 4) and follow by the procedures below:-

Step 5: Variant Calling for INDELs

% java -Xmx4g -jar $GATK -nt 4 -T UnifiedGenotyper $INPUT \

-R $REF \

-o $NAME.ug.raw.indel.vcf \

--genotype_likelihoods_model INDEL

Step 6: INDELs recalibration

% java -Xmx4g -jar $GATK -T VariantRecalibrator -R $REF \

-input $NAME.ug.raw.indel.vcf \

--maxGaussians 4 \

-std 10.0 \

-percentBad 0.12 \

-resource:mills,known=true,training=true,truth=true,prior=12.0 $MILLS \

-an QD \

-an FS \

-an HaplotypeScore \

-an ReadPosRankSum \

-an InbreedingCoeff \

-mode INDEL \

-recalFile $NAME.ug.indel.recal \

-tranchesFile $NAME.ug.indel.tranches \

-rscriptFile $NAME.ug.indel.plots.R

% java -Xmx4g -jar $GATK -T ApplyRecalibration -R $REF \

-input $NAME.ug.raw.indel.vcf \

--ts_filter_level 95.0 \

-tranchesFile $NAME.ug.indel.tranches \

-recalFile $NAME.ug.indel.recal \

-mode INDEL \

-o $NAME.ug.filter.indel.vcf

Intersection of indels of size within 50 bp discovered in CASAVA and GATK was the released indels set in SSIP. SNPEff was then used to annotate this consensus indels set.

**1.7 Structural Variation discovery (deletion)**

Four methods were used for the detection of large deletions in each sample: (i) BreakDancer v1.1._2011_02_21; (ii) VariationHunter Release_v0.3; (iii) Pindel version 0.2.2 and (iv) Delly v0.0.5.

The final set of deletions that are reported in the main text are union sets discovered by all four methods, and are between 50 bp and 10 Mbp in size. To evaluate the intersection of the deletions by four methods, we used PennCNV version 2011Jun16 available from [http://www.openbioinformatics.org/penncnv/penncnv_download.html#_Toc214817256](http://www.openbioinformatics.org/penncnv/penncnv_download.html#__RefHeading___Toc214817256) . “concordant” calls are defined as having a minimum reciprocal overlap of 50%. Breakpoints of a particular deletion were assembled base on the merged deletion with the longest length.

**1.7.1 BreakDancer deletion discovery**

BreakDancer adopts the paired-end mapping approach that utilizes paired-end sequencing reads to discover structural variants by relating the genomic coordinates of both ends of a paired-end read against the distribution of the insert sizes [37].

Input BAM files for BreakDancer exclude:

- QC failure reads;
- duplicated reads;
- reads with mapping quality < 35.

The bam2cgf.pl perl script was used to generate the input file, where we adopted the default option of 4 for the parameter on the standard deviation of the insert sizes. Workflow is as follow:

Step 1: Pre-processing raw bam files

% samtools view -q 35 -F 0x200 -b [sample].bam | samtools view -F 0x400 -b - > [sample].q35.bam

% bam2cfg.pl <sample>.q35.bam > [sample].q35.cfg

Step 2: Structural deletion calling

% breakdancer_max [sample].q35.cfg > [sample].q35.BD.SV

Step 3: Filtration

% more [sample].q35.BD.SV | awk '$9>25 && $10>2 && $7 == "DEL" && $1 == $4' > [sample].q35.BD.DEL

% ./remove_centromere.pl [sample].q35.BD.DEL > [sample].q35.BD.DEL.nogap

The raw output was subsequently filtered to minimize false discovery base on the following criteria:

- Minimum confidence score of 25;
- Supported by at least 3 reads;
- Both paired-end reads are mapped onto the same chromosome;
- Not located in the centromere.

**1.7.2 VariationHunter deletion discovery**

VariationHunter similarly adopts a paired-end mapping strategy based on maximum parsimony in identifying structural variants [38].

VariationHunter requires an input divet file which is converted from BAM with sam2divet.awk customized script, where we specified the *min* parameter to be the mean insert size minus four times the standard deviation of the insert sizes. The *max* parameter is set to the mean insert size plus four times the standard deviation of the insert sizes.

Only deletion calls meet the following criteria were retained:

- Supported by at least 3 distinct reads;
- Not located in the centromeres

The workflow is:

Step 1: Pre-processing raw bam files

% samtools view [sample].q35.bam | awk –f sam2divet.awk > [sample].q35.vh.divet

Step 2: Structural deletion calling

% echo [$min-200] [$max+200] 0 [sample].q35.vh.divet 2 | ./VariationHunter_SC > log &

Step 3: Filtration

%./remove_centromere_vh.pl [sample].q35.vh.divet.SV.Deletion > [sample].q35.vh.divet.SV.Deletion.nogap

**1.7.3 Pindel** **deletion discovery**

Pindel [39] is based on split-read analysis, it uses pattern growth approach in detecting structural variation from paired-end reads. The input file for Pindel was generated using the provided sam2pindel tool, using the following command line:

% samtools view [sample].bam | sam2pindel - [sample].pindel.txt [tag] [mean insert size] 0

The Pindel calling was done by:

% pindel. –f reference –p *pindel.txt –c ALL –o *.pindel.output

Filtration of Pindel called deletion was based on minimum of 10 supporting reads

% more [sample].pindel.output_D | awk '$2=="D" && $11-$10>10 {printf("%s\t%s\t%s\t%s\t%s\t1\n",$8,$10,$11,$11-$10,v1)}' v1=$file > $file.pindel.union

**1.7.4 Delly** **deletion discovery**

Delly adopts integrated approach of paired-end and split read analysis in discovering structural variation [40]. It calls deletion by using bam file of each sample with the assumption that each bam was separated by library or lane on the sequencer.

./delly –p –g ref.fa [sample].bam

Raw output of Delly was filtered according to sequence coverage, repetitiveness and insert size distribution. Only deletion with supporting reads of mapping quality at least 20 and within length of 50bp to 10Mbp were retained. Filtering was performed using:

% grep '>Deletion*' [sample].out | awk '$6>=20 && $4>50 && $4<10000000 {printf("%s:%s-%s numsnp=3 length=%s state2,cn=1 Delly_raw startsnp=rs123 endsnp=rs321 conf=%s\n",$1,$2,$3,$4,$5)} '

**1.8 Haplotype phasing of genotype data**

The set of consensus bi-allelic SNPs detected by both CASAVA and GATK was considered for phasing, where we used the genotype calls made by CASAVA. Phasing was performed using BEAGLE 3.3.2 (<http://faculty.washington.edu/browning/beagle/beagle.html>) with the following command line:

% java –Xmx 10000m –jar beagle.jar unphased=chr*.bgl missing=0 out=chr*

In house customized script was used to convert the phased output to variants file VCF format.

**1.9 Mitochondria haplogroup assignment**

Haplogroup assignment of mitochondria was performed using customized script and online tool haplogrep [19, 45], the first step involved generation of consensus FASTA file of complete mitochondria sequence by SAMTOOLS’s mpileup and GATK’s FastaAlternateReferenceMaker. The generated FASTA file was converted to .hsd file as an input data to haplogrep that was ran with default settings. Procedures involved in assigning mitochondria haplogroup of each individual are as follow:

Step 1: Generation of VCF from BAM file

% samtools mpileup –E –q30 –Q30 –C50 –r MT:1-16569 –uf <rCRS.fasta> chrM.bam | bcftools view –cg - > output.vcf

Step 2: Assignment of ‘N’ to heterozygous calls by using LINUX command line

Step 3: Generation of FASTA file

% java –jar GenomeAnalysisTK.jar –T FastaAlternateReferenceMaker –R <rCRS.fasta> -o output.fasta –variant output.vcf

Step 4: Creation of input file base on format required for haplogrep by using in-house Python script

% python fas2hsd.py output.fasta

Step 5: Online submission of query to haplogrep at <http://haplogrep.uibk.ac.at/>

**1.10 Chromosome Y haplogroup assignments**

To assign haplogroup for chromosome Y of male samples in SSIP, in-house script was prepared to generate necessary input file to haplogroup assignment software YFitter v0.2 base on 2008 Y tree [47].

VCF file was created to include homozygous reference positions and the file was converted to qcall format using customized Phyton script. qcall file serves as input to YFitter tool for haplogroup assignment. Briefly, Yfitter with default parameters scans through a list of chromosome Y positions to assign haplogroup, the command used:

% ./Yfitter –m karafet_tree_b37.xml file.qcall

**4 References**

1. Quinlan AR, Hall IM (2010) BEDTools: a flexible suite of utilities for comparing genomic features. Bioinformatics 26: 841-842.

2. Wang K, Li M, Hadley D, Liu R, Glessner J, et al. (2007) PennCNV: an integrated hidden Markov model designed for high-resolution copy number variation detection in whole-genome SNP genotyping data. Genome Res 17: 1665-1674.
